# Scorpiand Pyrazole Azacyclophanes: Synthesis, Protonation Behavior, and Cu^2+^ Coordination Chemistry

**DOI:** 10.1021/acs.inorgchem.6c02243

**Published:** 2026-08-27

**Authors:** Irene Bonastre-Sabater, Alberto Lopera, Salvador Blasco, José M. Llinares, Mario Inclán, Antonio Doménech-Carbó, Enrique García-España, M. Paz Clares

**Affiliations:** † Departamento de Química Inorgánica, Instituto de Ciencia Molecular, Universidad de Valencia, C/Catedrático José Beltrán 2, Paterna (Valencia) 46980, Spain; ‡ Departamento de Química Orgánica, Facultad de Farmacia y Ciencias de la Alimentación, Universidad de Valencia, Av. Vicent Andrés Estellés, n° 22. Burjassot (Valencia) 46100, Spain; § Departamento de Farmacia, Facultad de Ciencias de la Salud, Universidad CEU Cardenal Herrera, Lluís Vives, 1, Alfara Del Patriarca, (Valencia) 46115, Spain; ∥ Departamento de Química Analítica, Facultad de Química, 16781 Universitat de València, Av. Vicent Andrés Estellés, 19C. Burjassot (Valencia) 46100, Spain

## Abstract

We report the synthesis, protonation, and Cu^2+^ coordination properties of a family of scorpiand ligands based on a 3,6,9-triaza-1­(3,5)-1*H*-pyrazolacyclodecaphane scaffold bearing aminoethyl, pyridyl, or quinolyl side arms. Potentiometric, UV–vis, and NMR studies reveal three protonation steps for L1 and four for the pyridyl- and quinolyl-substituted ligands, owing to the additional protonation of the heteroaromatic donor. Cu^2+^ coordination leads predominantly to mononuclear complexes, whereas L1 also forms binuclear and trinuclear species. The relatively low stability constants of the [CuL]^2+^ complexes, compared with related pyridinacyclophanes, indicate coordination by a limited number of nitrogen donors. Single-crystal X-ray diffraction of [Cu­(H**L1**)­Br_2_]­Br·H_2_O confirms a tridentate coordination mode involving the side-chain amine, one secondary amine, and the tertiary amine of the macrocycle, with the pyrazole ring adopting a noncoordinating exo orientation. Spectroscopic studies show that protonation controls the side-chain dynamics of the free ligands, whereas Cu^2+^ binding locks the ligand into a more rigid conformation. The resulting coordination environment affords highly efficient superoxide dismutase mimics, with catalytic activities among the highest reported for related systems.

## Graphical abstract

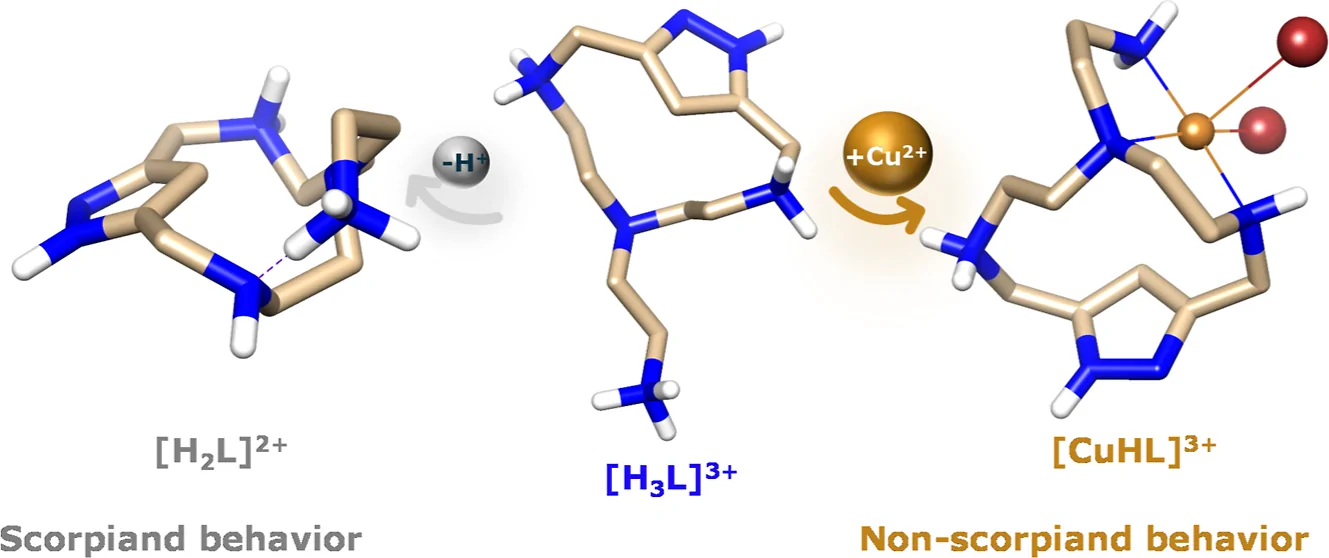

## Introduction

Scorpiand-type azamacrocycles were synthesized for the first time by the Swiss chemist T. Kaden, condensing diacetylpyridine with *N,N*-*bis*(3-aminopropyl)-*Ń,Ń*-dimethylenediamine using Ni^2+^ as a template, followed by hydrogenation to obtain the Ni^2+^ complex.^1,2^ The nickname “scorpiand” was coined by the Italian chemist L. Fabbrizzi to address the movement that the pendant arm of the Ni^2+^ complexes of the macrocycle *N*-(aminoethyl)­cyclam experiences with pH or with an oxidation process.^3^ In the Ni^2+^ complexes at low pH, the primary amine at the “scorpiand” tail is protonated and lies as far apart as possible from the metalated macrocyclic core in order to minimize electrostatic repulsions, while at higher pH values, the pendant arm folds toward the macrocycle core to allow the coordination of the nitrogen atom in the “scorpiand” tail to the metal cation, to complete its coordination sphere. Oxidation of the Ni^2+^ complex to Ni^3+^ also favors the formation of the closed conformation with the amine at the tail coordinated to the metal ion. In this respect, Fabbrizzi and co-workers have developed several “scorpiand-type” complexes based on the well-known structure of cyclam as the macrocyclic core, and they have studied how the in-out movement of the coordinated tail was regulated by different chemical and electrochemical inputs.^4,5^ An interesting scorpiand-type phenanthroline azamacrocycle containing an anthracene group has also been reported, in which the conformational change was induced by coordination to Zn^2+^.^6^

A few years ago, our team developed scorpiand ligands based on the macrocyclic core 3,6,9-triaza-1­(2,6)-pyridinacyclodecaphane, hereafter py22,^7,8^ with different 2-aminoethyl derivatives connected to the central nitrogen of the polyamine bridge. The parent compound of this series was prepared by reacting the tosylated tripodal polyamine *tris*(2-aminoethyl)­amine (tren) with 2,6-*bis*(bromomethyl)­pyridine, followed by purification by crystallization and removal of the tosyl groups.^9^ Once detosylated, the primary amine at the pendant arm can be easily derivatized by reaction with an aldehyde to give the Schiff base, which is further reduced with sodium borohydride. Following this strategy, different aryl units have been appended to the side chain to explore how its molecular motion affects the chemical, physical, or even biological properties of the free ligands and their metal complexes. For example, it was shown that in the compounds having naphthalene or anthracene groups at the pendant arm, the motion could be switched ON or OFF only by changes in the pH, in the absence of metal ions. At sufficiently acidic pH values, when the three secondary amine groups are protonated, the ligand acquires the extended open conformation, while as soon as the first proton is lost, it switches to the closed conformation favored by hydrogen bond formation between the protonated and nonprotonated amines as well as π–π stacking between the aryl group at the tail and the pyridine of the core. Moreover, studies regarding the interaction of scorpiand ligands having extended aromatic rings in the pendant arm with calf thymus DNA showed that the binding mode was regulated by metal ions: at neutral pH and in the absence of metal ions, the scorpiand ligands adopt an extended conformation, allowing the aryl group to intercalate between the base pairs of DNA. On the other hand, coordination to metal ions forces the closed conformation, impeding the intercalation from occurring, and this is translated into a much higher toxicity of the free ligands in cells.^10^a Also, ligands with this topology have been used for the fluorescent discrimination of nucleic bases sequences,^10b^ and to build antiparasitic and antimicrobial agents.^11^ Interestingly, the type of coordination offered by the heterocyclic rings in the tail may modulate the antioxidant activity of coordinated electroactive metal complexes; complexes of ligands with 4-quinoline substituents at the tail present higher superoxide dismutase (SOD) and anti-inflammatory activity than those with 2-quinoline in which coordination to the metal of the heterocyclic nitrogen occurs.^12^ At the same time, tail-tied double aza-scorpiand ligands have shown an interesting mimicry behavior of the Cu_2_Zn_2_-SOD enzyme.^13^ Derivatization of the pendant arm with a 6-amino-3,4-dihydro-3-methyl-5-nitroso-4-oxopyrimidine group produced systems prone to be attached to the electron-reach surface of carbon nanotubes leading to supported ligands able to capture and remove metal ions, or behave as heterogeneous catalyst as for the Cu-free Sonogashira coupling reaction in the case of Pd^2+^ complexes, with excellent performance in terms of yields and reaction times, and unprecedented ability to work under environmentally friendly conditions, such as in water at 50 °C and in an aerobic atmosphere.^14^

In view of this scenario, it seemed interesting to synthesize scorpion-like ligands with macrocyclic cores containing other heterocycles. Taking into account our previous work on [1 + 1], [2 + 2], and [3 + 2] condensation polyaza macrocycles of 1*H*-pyrazole, and due to the many binding possibilities that this spacer may offer, we decided to introduce this heterocyclic unit in the scorpiand core.^15,16^ Figure 1 schematically shows examples of the previous scorpiands and the family of compounds presented in this work.

**Figure 1:**
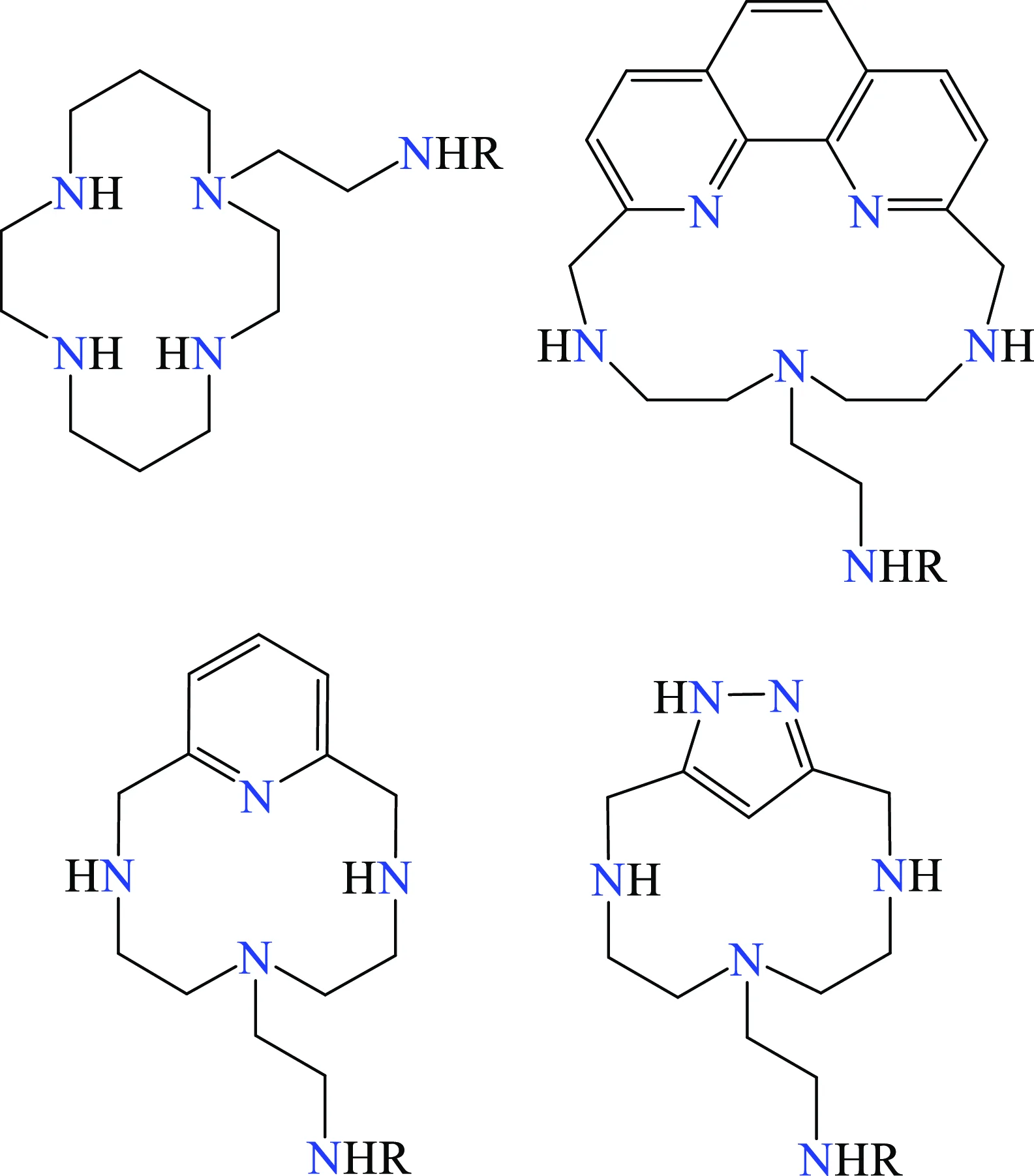
Schematic representation of previous azascorpiand ligands and current compounds (refs 1–6 and 9–13 ).

Indeed, depending on the length of the polyamine chain in the [1 + 1] macrocycles, the pyrazole can have either endo- or exocoordination, giving rise to discrete monomeric or dimeric complexes with a variety of stoichiometries and topologies. In the case of [2 + 2] and [3 + 2] condensation ligands, monomeric binuclear complexes are normally obtained. However, by controlling the pH and selecting appropriate macrocycles and cryptands, particularly metallocages or double-helical structures, can be obtained.^16^ ^e^

In this work, we report that, starting from the macrocycle resulting from the condensation of tosylated tris­(2-aminoethyl)­amine and 3,5-*bis*(chloromethyl)-1*H*-pyrazole and the removal of the tosylated groups (pztren, **L1**), we have prepared two series of isomeric compounds: one with pyridine substituents in the tail (pzt2py, pzt3py, pzt4py), **L2**-**L4**), and the other with quinoline substituents (pzt2q, pzt4q, **L5**-**L6**) (Figure 2).

**Figure 2:**
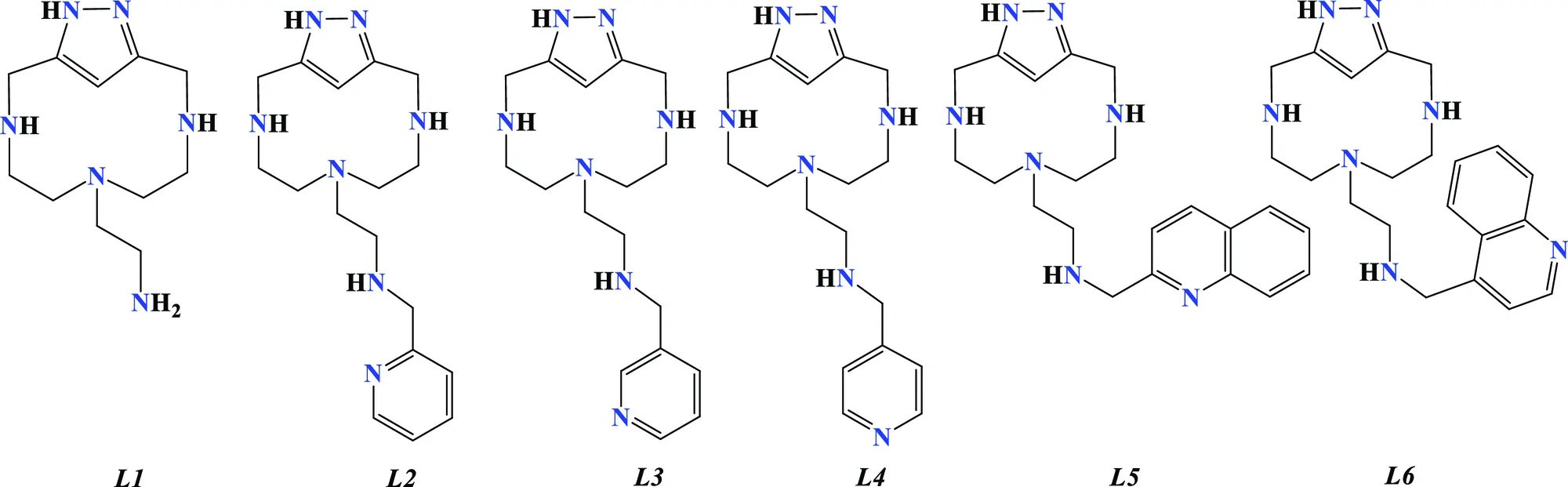
Schematic structures of **L1**-**L6**.

In this article, in addition to the synthesis, we present relevant aspects of their Cu^2+^ coordination both in solution and in the solid state and provide initial evidence of the potential of these compounds to remove radical species. We demonstrate how replacing the pyridine heterocycle in the macrocyclic core with 1*H*-pyrazole completely modifies its coordination chemistry while increasing the antioxidant capacity of the Cu^2+^ complexes.

## Results and Discussion

### Synthesis of the “Scorpiand” Ligands

The synthesis of **L1**-**L6** was carried out using a modification of the Richman–Atkins procedure^17,18^ already employed for obtaining the analogous pyridine scorpiands.^9^ **L1** was synthesized by reacting under reflux the protected pyrazole ring 3,5-*bis*(chloromethyl)-1-(tetrahydropyran-2-yl)-pyrazole with the pertosylated tris­(2-aminoethyl)­amine (tren) in a 1:1 molar ratio in dry CH_3_CN using K_2_CO_3_ as a base. The protection of the pyrazole group is necessary to avoid side reactions.^19^ The cyclization step was followed by the removal of the protecting groups in an acidic medium using phenol and a HBr/HAcO acid mixture. **L2**-**L6** were obtained by reacting **L1** in its free-amine form with their corresponding pyridine and quinoline carbaldehydes in dry ethanol, followed by *in situ* reduction of the corresponding imine with sodium borohydride. The compounds were finally isolated as hydrobromide or hydrochloride salts (Scheme 1).

**Scheme 1:**
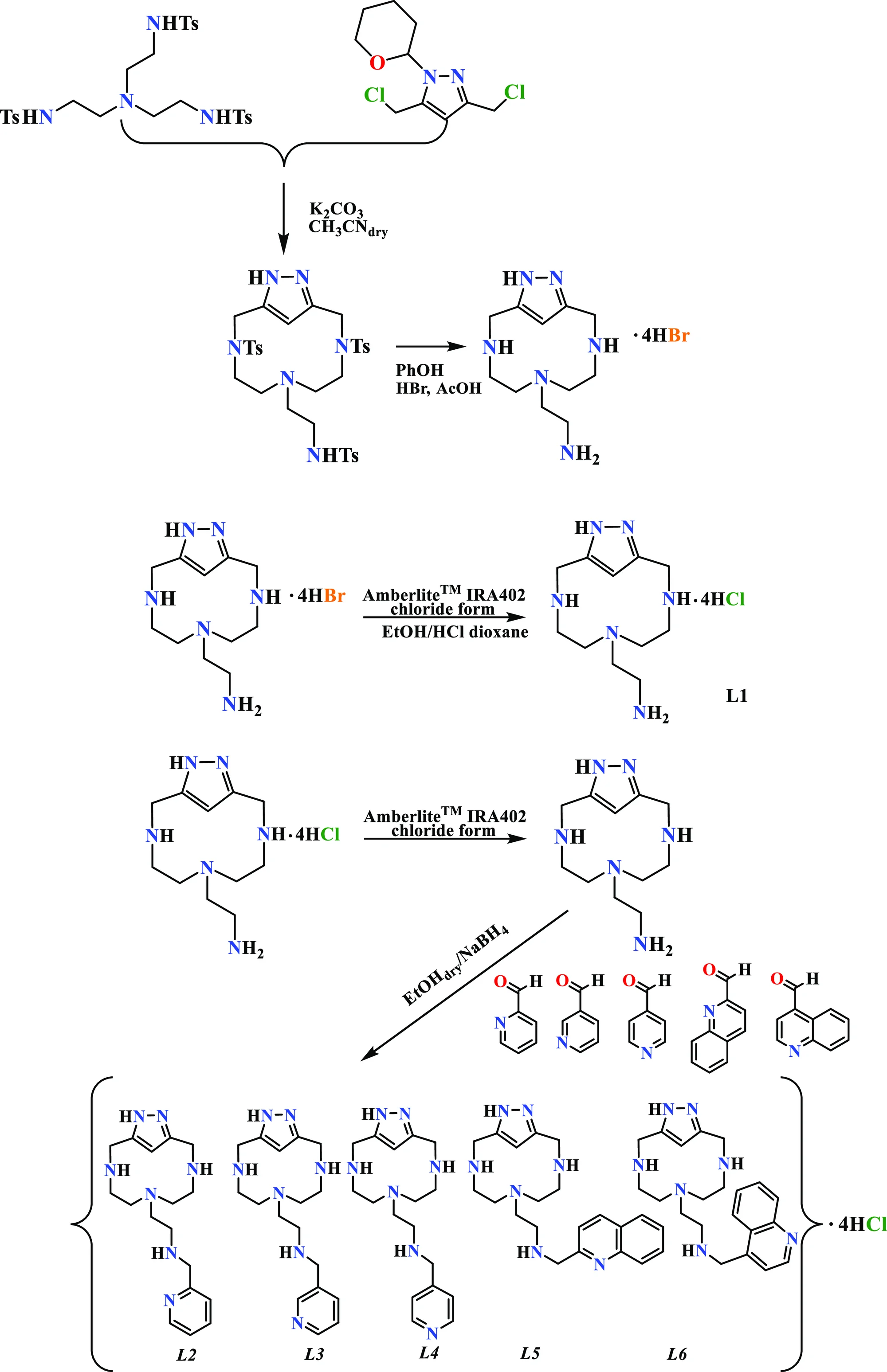
Synthetic Scheme for Obtaining **L1**-**L6**

### Acid–Base Behavior

Table 1 presents the stepwise protonation constants derived from the cumulative basicity constants determined from the pH-metric titrations using the HYPERQUAD set of programs.^20^ Distribution diagrams for selected systems calculated with the logician HySS^21^ are shown in Figures 3, 7, and 8, and the remaining ones are given in the Supporting Information (Figure S1).

**Table 1:** Logarithms of the Stepwise Protonation Constants of **L1**-**L6** Measured at 298.1 ± 0.1 K in 0.15 M NaCl

reaction^a^	L1	L2	L3	L4	L5	L6
L + H ⇌ HL	9.45(1)^b^	9.20(1)	9.13(2)	10.01(3)	9.13 (1)	9.06 (2)^b^
HL + H ⇌ H_2_L	8.63(1)	7.90(1)	7.96(1)	8.03(4)	7.84 (1)	7.58 (2)
H_2_L + H ⇌ H_3_L	7.03(1)	6.77(1)	6.64(2)	6.48(6)	6.60 (1)	6.29 (2)
H_3_L + H⇌ H_4_L	-	2.97(1)	3.40(2)	3.46(8)	2.35 (3)	3.18 (2)
log β^c^	25.12(2)	26.84(2)	27.12(5)	29.38(8)	25.92 (4)	26.12 (7)

a Charges omitted.

b Numbers in parentheses are standard deviations in the last significant figure.

c Calculated as log β = Σlog K_HjL_.

**Figure 3:**
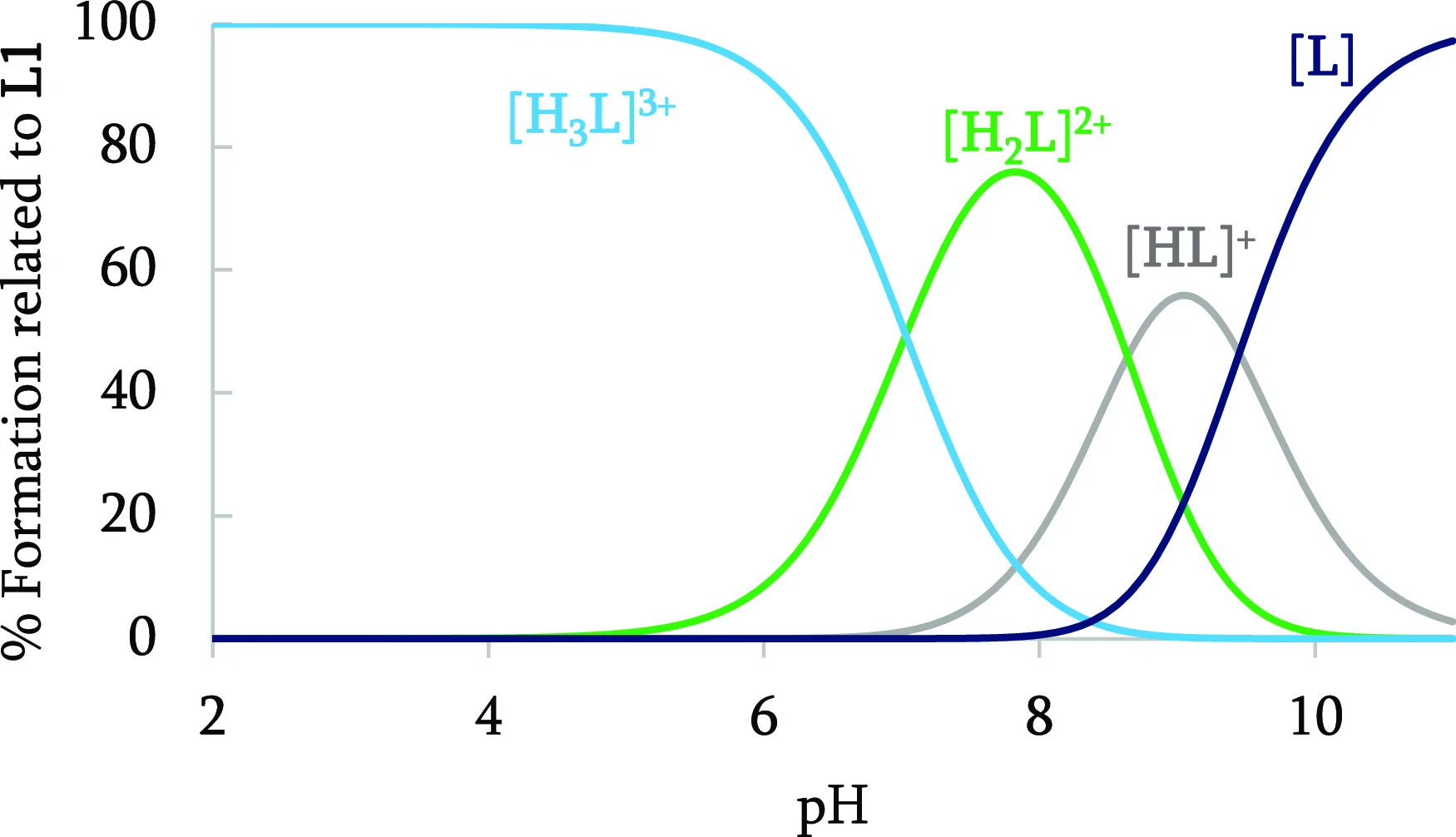
Distribution diagram for the protonation of **L1**.

**L1** exhibits only three protonation steps within the pH range accurately accessible to pH-metric measurements (pH = 2.5–11.0); these can in principle be attributed to the protonation of the primary amine in the side chain and the secondary amine groups of the macrocyclic core. In contrast, all other compounds display four protonation steps, which can be initially assigned to the protonation of the three secondary amine groups and the nitrogen atom of the heterocycle in the pendent arm. It seems that, as already described for similar systems, in none of these compounds does protonation or deprotonation of the 1*H*-pyrazole unit occur.^16,23–25^ In fact, processes affecting pyrazole occur at very basic or very acidic pH values, which are clearly outside the pH range available for potentiometric studies.^22,26^

The first three constants show, for all of the ligands, a progressive decrease due to statistic effects and electrostatic repulsions between charged amino groups.^27,28^ The much less basic fourth constants show increasing values as the substitution changes from the *ortho*- to *para*-position in the pyridine ring and from the 2- to 4-position in the quinoline. On the other hand, the values of the stepwise protonation constants are, in general, a little bit lower than those we have previously obtained for related pyridinaazacyclophane scorpiands.^9^

In order to find out a plausible protonation pattern and validate our initial guess, we have recorded the pH-dependent variation of the UV–vis and ^1^H and ^13^C NMR spectra for selected systems; all NMR signals were assigned accordingly to different 2D mono- and heteronuclear correlation experiments. At pH 2, **L1** shows a band centered at 203 nm that slightly shifts bathochromically and increases in intensity as the pH rises and the macrocycle deprotonates (Figure S2). This behavior suggests an involvement of the pyrazole unit in this process, likely through hydrogen bonding (vide infra).

The ^1^H NMR spectra of **L1** (Figure 4) show, at low pD values, where the triprotonated species [H_3_L]^3+^ prevails in solution, singlet signals for the pyrazole proton Hpz4 and for the protons of the methylene group closest to the 1*H*-pyrazole spacer, H1. All of the other protons appear as well-defined triplet signals, except for H3, which presents a very broad signal that is essentially masked within the baseline. Until pD 6, no noticeable change in the chemical shifts is observed (Figure S3). Then, upon increasing the pD to 7.43, in correspondence with the deprotonation of the first ammonium group, the signals of protons H1, H2, and also of Hpz4 shift upfield in correspondence with the first deprotonation process.^29^ At the same time, the signal of protons H3 starts to emerge from the baseline as a very broad resonance. The second deprotonation process, which is completed at pD = 9.43, leads to further upfield shifts of the H1, H2, and Hpz4 signals. At this pD, the signal of proton H3 is clearly visible as a broad signal centered at 1.87 ppm. At higher pD values, the most pronounced upfield shift is observed for H5, while the signal of H3 remains visible but does not show any significant change in the chemical shift.

**Figure 4:**
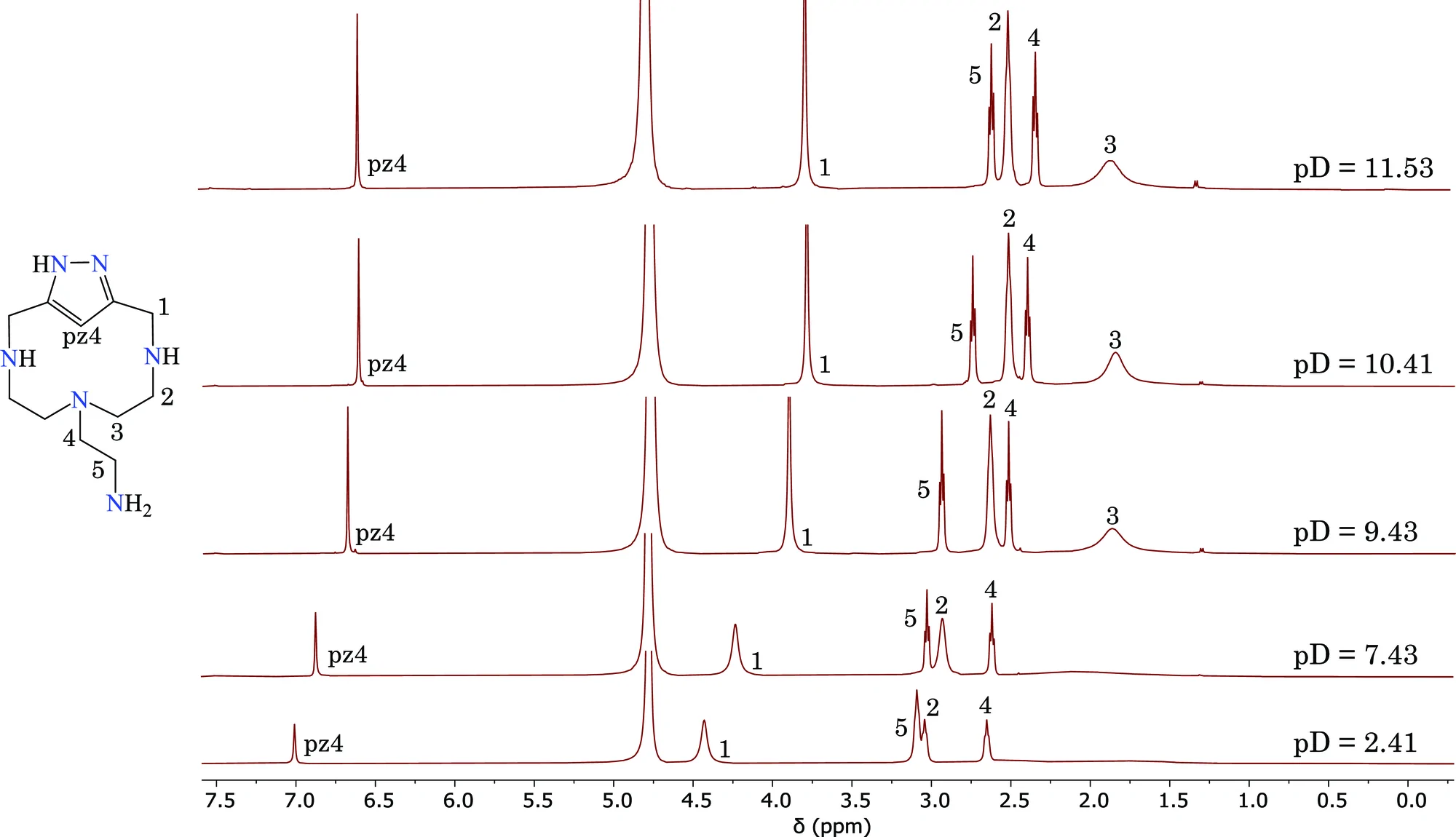
Variation of the ^1^H RMN of **L1** with the pH recorded in D_2_O.

These observations suggest an average protonation pattern in which the primary amine of the pendant arm protonates first, followed by the two secondary amine groups of the macrocyclic core. Additionally, the data indicate that the tertiary amine does not become protonated within the explored pH range. On the other hand, the appearance/disappearance of the signals of protons H3 may denote dynamic processes involving the side arm.

The changes observed in the ^13^C NMR spectra further support the conclusions mentioned above. As is well established, the ^13^C signals most affected by the deprotonation of nearby amine groups are those placed at the β-position with respect to them, which undergo downfield shifts.

As the first proton leaves the molecule ongoing from pD 2.41 to 7.43, the signal shifting downfield most is that of C3 (Δδ = 1.2 ppm), which is located in the β-position with respect to the secondary amine groups of the macrocycle (Figure 5). Increasing further the pD to 9.43 leads to an additional downfield shift of 1.8 ppm for this resonance. Upon further increasing the pD to 10.41, significant downfield shifts are observed for signal C4, placed at the β-position relative to the primary amine of the side chain. This signal undergoes a downfield shift of Δδ = 2.3 ppm from pD 9.43 to 10.41 and another one of 1.8 ppm from pD 10.41 to 11.53.

**Figure 5:**
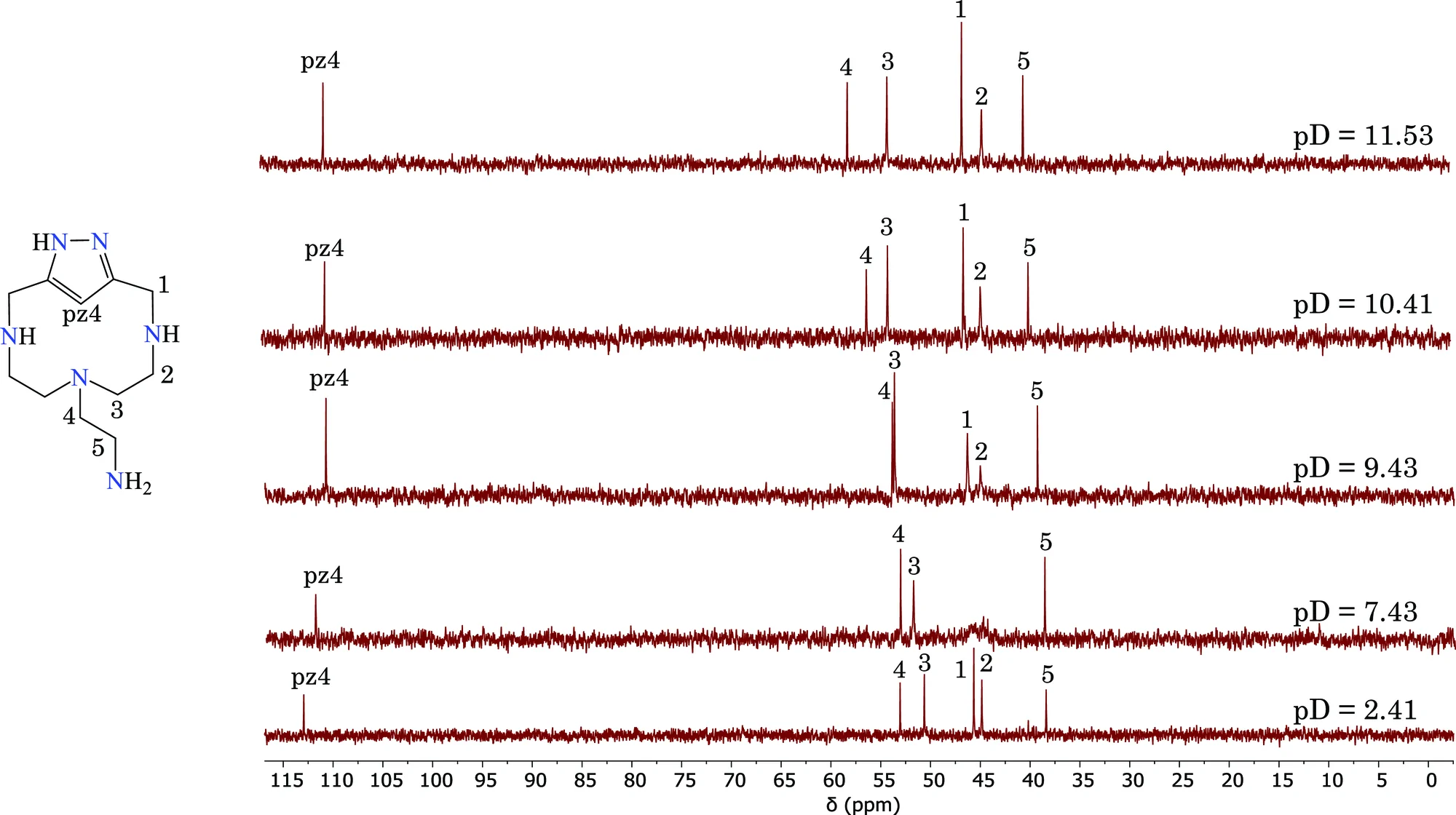
Variation of the ^13^C RMN of **L1** with the pH recorded in D_2_O.

These observations are fully consistent with the average protonation pattern derived from the ^1^H NMR data, which indicates the initial protonation of the primary amine of the pendant arm, followed by protonation of the secondary amine groups of the macrocyclic core. No evidence is observed for the protonation of either the tertiary amine or the pyrazole ring. Indeed, it is well-known that primary amine groups are more basic in aqueous solution than secondary ones primarily due to their more favorable hydration; a primary ammonium can form up to three hydrogen bonds with surrounding water molecules, enhancing its stability.

Another point that is particularly noticeable is the appearance and disappearance of selected ^1^H and ^13^C NMR signals at given pH values. To gain more insight into this dynamic behavior, variable-temperature ^1^H NMR and ^13^C NMR spectra were recorded at pD = 7.43 from room temperature and 353 K (Figures 5 and S4).

The ^1^H NMR shows that the signal of proton H3 becomes observable as the temperature increases and exhibits slight narrowing, even though it remains broad even at 353 K (see Figure S4). A similar behavior is observed in the ^13^C NMR spectra, but in this case, the signals attributed to carbon atoms C1 and C2, which are closest to the secondary amine groups, are initially overlapped with the baseline and only appear as sharp peaks at 353 K. Notably, upon cooling the samples back to 298 K, the spectra recovered their original characteristics, including the disappearance of these signals (Figure 6).

**Figure 6:**
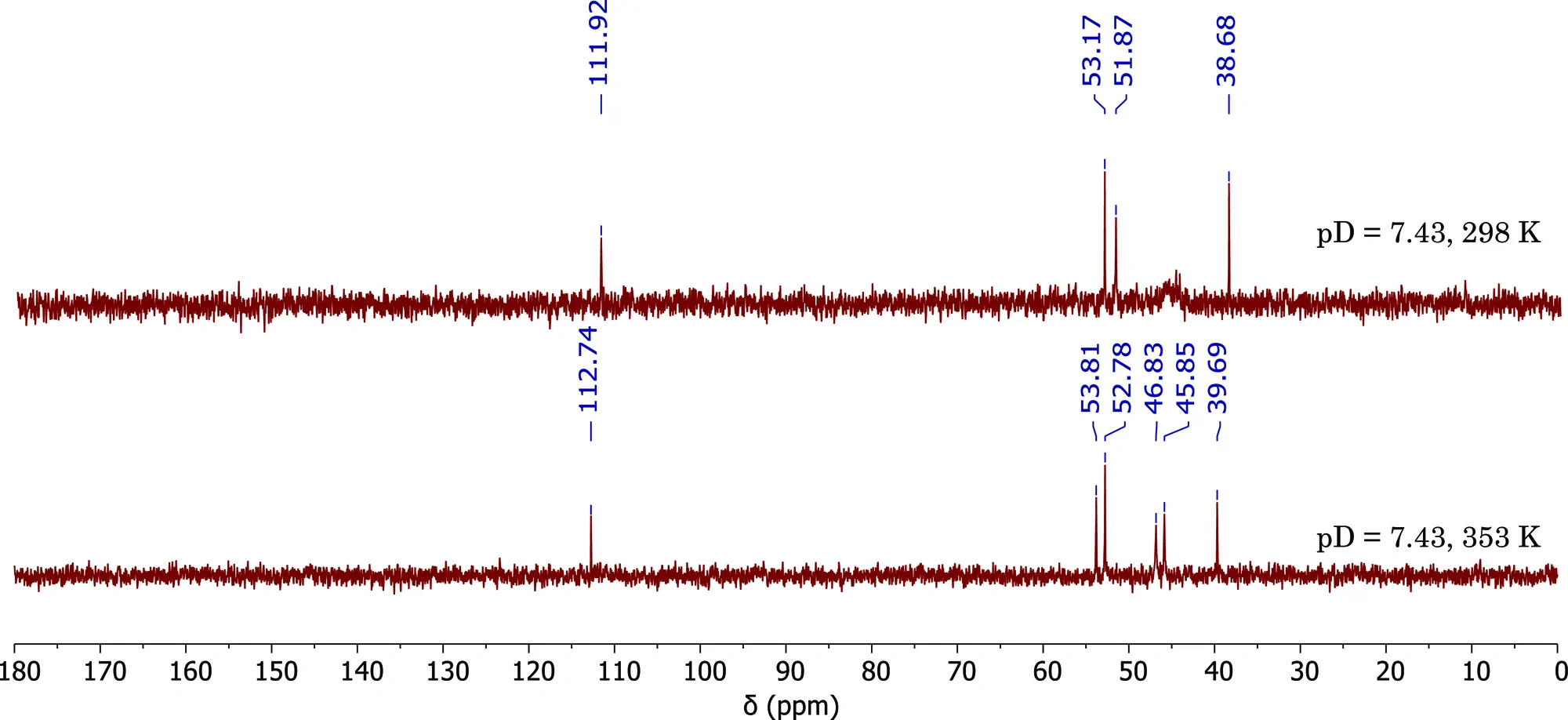
^13^C NMR spectra of **L1** at 298 K (top) and 353 K (bottom) at pD 7.43.

A plausible interpretation is related to the movement of the pendant arm toward or outward from the macrocyclic core, leading either to closed or extended conformations. As we have previously described for related pyridinacyclophanes bearing aryl substituents in their pendant arms,^9,10^ protonation of the three secondary amine groups forces the compound to adopt an extended open conformation in which the positively charged ammonium groups are positioned as far apart as possible to minimize electrostatic repulsions. As soon as the macrocycle is deprotonated, the flexible pendant arm bends toward the macrocyclic core. However, unlike scorpiand ligands containing pyridine in the macrocyclic core,^9^ the present pyrazole-based scorpiands do not require an aryl group in the pendant arm to undergo the bending motion. Therefore, in this case, the conformational change appears to be driven solely by hydrogen bond formation, without the need for π-stacking interactions between aromatic units. These hydrogen bonds may involve either the secondary amine groups of the macrocycle or the nitrogen atoms of the pyrazole ring, which would explain the dynamic NMR behavior observed, consistent with a slow exchange within the NMR time scale.

UV–vis and ^1^H NMR spectra at variable pH were also recorded for **L3**. The UV spectra show, in this case, two bands centered at about 210 and 260 nm, due to the absorption of the 1*H*-pyrazole and pyridine units, respectively. As can be seen in Figures 7 and S5, these two bands show opposite trends with pH, while the intensity of the band at 260 nm decreases with pH, reaching a plateau after the first protonation step; the band at 210 nm starts to slightly increase its intensity after the first deprotonation.

**Figure 7:**
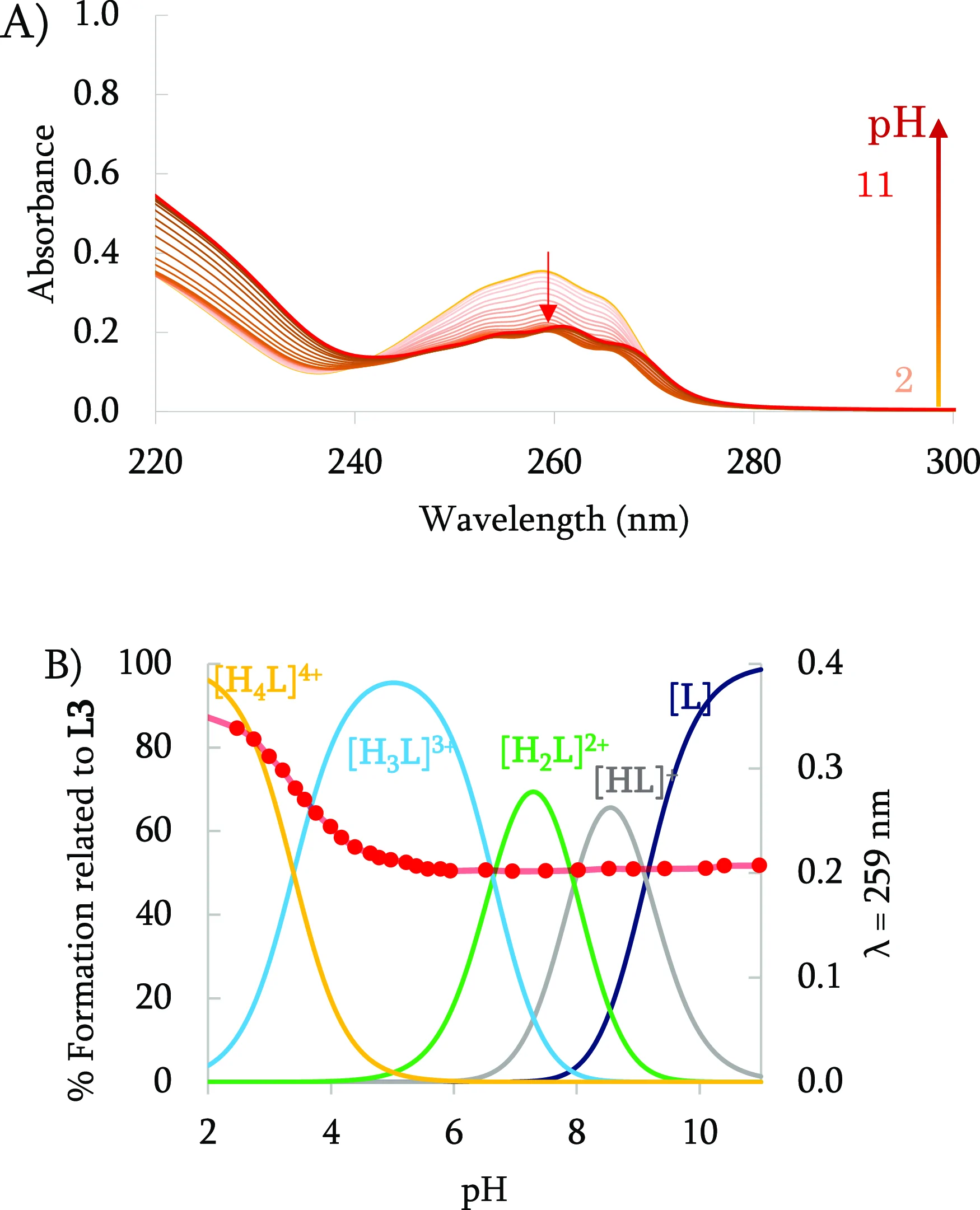
A) Variation of the UV–vis spectra of **L3** with pH. (B) Variation of the band centered at 259 nm with pH overlapped with the distribution diagram. [L] = 1 × 10^–4^ M.

The first characteristic indicates that, as suggested above, protonation of the pyridine ring in the side chain occurs in correspondence with the fourth protonation step. In fact, by processing the UV data with the HypSpec^20^ program, it was possible to obtain a log K value of 3.37(1), which is consistent with the value derived from pH potentiometric titrations (log K = 3.40, see Table 3). The ^13^C NMR spectra for this system (Figure S6) show a pattern similar to that of **L1**, but in this case, the further protonation of the nitrogen of pyridine produces significant changes in the aromatic signals below pH 4.

We have also recorded the variations in the UV–vis and ^1^H NMR spectra with the pH for **L6**, which is functionalized with a quinoline in position 4. At acidic pH, the UV spectra display, besides the absorption of pyrazole below 220 nm, two absorption bands centered at about 230 and 320 nm (Figures 8 and S7). When the pH is increased, two isosbestic points are observed at 229 and 299 nm. By processing the data with the HypSpec^20^ program, a constant of 3.69(1) logarithmic units is obtained, which could correspond to the protonation of quinoline and, again, is consistent with the potentiometric values.

**Figure 8:**
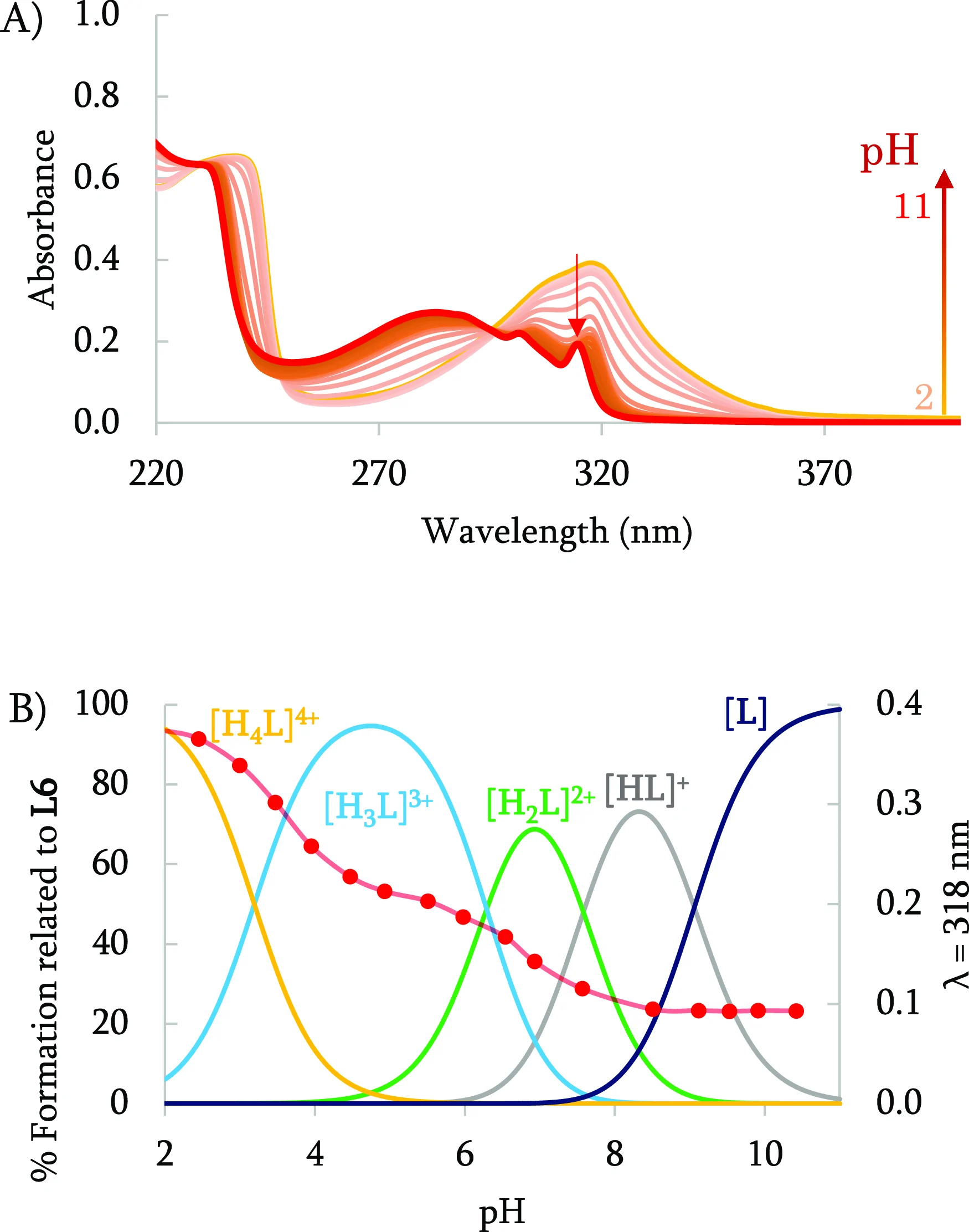
A) Variation of the UV–vis spectra of **L6** with pH. (B) Variation of the band centered at 318 nm with pH overlapped with the distribution diagram. [L] = 1 × 10^–4^ M.

### Molecular Dynamics Calculations

To rationalize the results obtained in the NMR titration experiments (i.e., the splitting and broadening of the signals following the deprotonation of the ligand at increasing pH values), molecular dynamics calculations were performed for **L3** with different protonation degrees. For the conditions to be as similar as possible to the experimental ones, the models were built using explicit solvent (10 Å TIP3P water box) and Cl^–^ as counterions. Selected results are shown in Figure 9, and the movies of the full 3 ns simulations can be downloaded from the Electronic Supporting Information.

**Figure 9:**
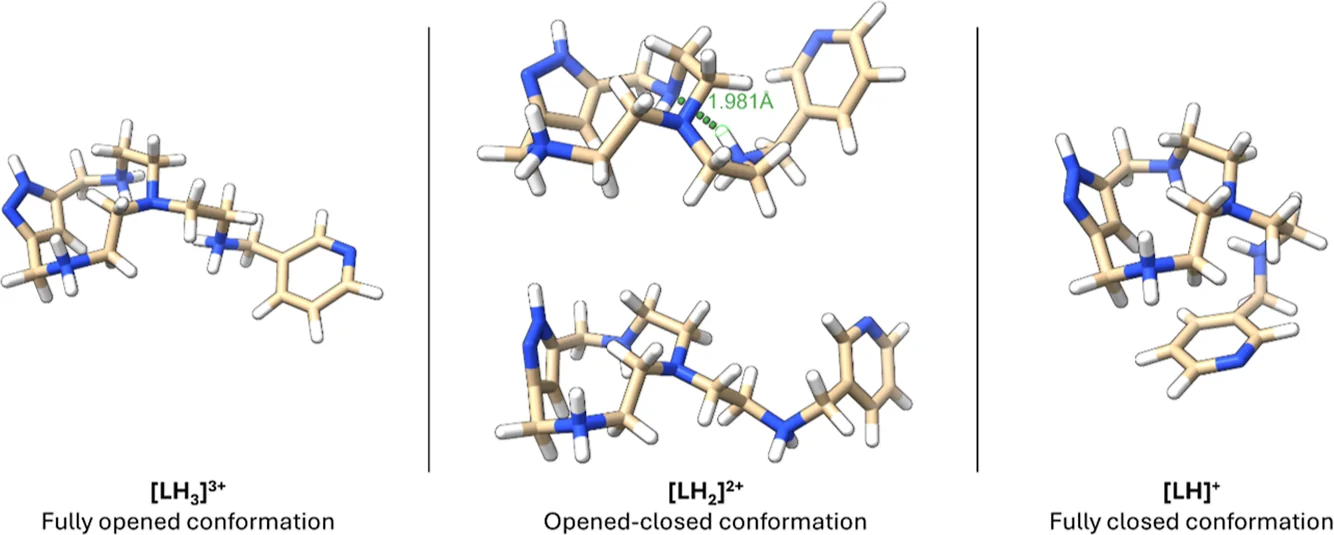
Representative ligand conformations obtained in the molecular dynamics simulations of [H_3_L]^3+^ (left), [H_2_L]^2+^ (center), and [HL]^+^ (right) species. Performed using GAFF potentials and explicit solvent (TIP3P water and Cl^–^). A video is included in the Supporting Information.

For the fully protonated ligand, [H_3_L]^3+^, an “open” conformation, with the side chain positioned away from the macrocycle, was found to be maintained throughout the simulation. This rigid conformation is kept in place by the strong electrostatic repulsion between the three positively charged ammonium groups. This is a highly symmetrical and stable structure that correlates well with the well-defined NMR spectra registered at low pH values.

The situation changes drastically after the first deprotonation step. In the simulation of [H_2_L]^2+^, a fluxional behavior is observed, with the side chain now alternating between the open conformation and a closed one, with the ammonium group of the side chain forming hydrogen bonds with the amino group of the macrocycle.

In the last simulation performed for the [HL]^+^ species, the closed conformation becomes predominant. Since the electrostatic repulsions have now disappeared, the polar contacts between the pendant arm and the macrocycle now prevail, keeping the ligand in this bulky conformation throughout the simulation. This structure, although more rigid than the previous step, is still unsymmetrical, with the side chain positioned toward one of the sides of the macrocycle.

### Cu^2+^ Coordination Chemistry

The interaction of the scorpiand ligands **L1**-**L6** with Cu^2+^ was first analyzed by pH-metric titrations to determine the stability constants. Complexes of [Cu­(H_*x*_L)]^(2+x)^ stoichiometry with −1 ≤ *x* ≤ 1 were detected for **L1**, with −1 ≤ *x* ≤ 2 for **L2**, **L3,** and **L6** and with −2 ≤ *x* ≤ 2 for **L4** and **L5**. Distribution diagrams are shown in Figure S8. The stability constant of the [CuL]^2+^ complexes is markedly lower than that displayed by the corresponding azapyridinaphane ligands. For example, pytren,^9^ the analogous ligand to **L1**, for which penta-coordination of Cu^2+^ was ascertained both in solution and the solid state has a value of the stability constant of 20.43 logarithmic units, and pytren3py,^9^ ^b^ which behaves as a tetradentate ligand has a constant of 17.68 logarithmic units, suggesting that neither the nitrogen atom of pyridine nor the amine group in the pendant arm were involved in the coordination. In the case of the Cu^2+^ complexes of pytren2py, for which the involvement of all six nitrogen atoms present in the ligand was evidenced both in solution and in the solid state, the value of the stability constant for [CuL]^2+^ was 22.6 logarithmic units. Comparing with related systems, synthesized by us or found in the literature,^8,9^ the obtained stability constants suggest that the number of coordinated nitrogen atoms should be 3, except for the ligand with the *ortho*-substituted pyridine **L2,** which probably has 4 coordinated nitrogen atoms. However, as differences in protonation constants may lead to an erroneous interpretation of stability, we have calculated the pCu values for [L] = 1 × 10^–5^ M and [Cu^2+^] = 1 × 10^–6^ M^30^ (Table 2). The resulting pCu values are consistent with the stability constants, confirming that the most stable complexes within each family are those bearing the substituent at the 2-position, namely, L2 and L5, with pCu values of 13.8 and 11.5, respectively. The pCu-pH plots shown in Figure S9 further indicate that complexation is essentially quantitative above pH 7 for all the systems.

**Table 2:** Logarithms of the Stability Constants for the Cu (II)-**L1** to Cu (II)-**L6** Systems, Determined in 0.15 M NaCl at 298.1 ± 0.1 K

reaction^a^	L1	L2	L3	L4	L5	L6
CuHL + H ⇌ CuH_2_L	^–^	3.93 (2)	4.20 (4)	4.50 (4)	4.16 (2)	4.34 (4)^(^ ^b^ ^)^
CuL + H ⇌ CuHL	6.06 (1)^b^	6.02 (2)	5.895 (8)	5.53 (2)	5.92 (2)	5.91 (2)
Cu + L ⇌ CuL	12.20 (1)	15.35 (2)	11.35 (7)	12.70 (1)	12.86 (2)	10.87 (2)
CuL ⇌ CuH_–1_L + H	–8.13 (2)	–10.65 (3)	–8.38 (1)	–7.32 (2)	–9.32 (2)	–8.40 (5)
CuH_–1_L ⇌ CuH_–2_L + H	–10.45 (5)	-	-	–9.66 (4)	–11.14 (4)	-
2Cu + L ⇌ Cu_2_H_–2_L + 2H	1.76 (3)	-	-	-	-	-
Cu_2_H_–2_L ⇌ Cu_2_H_–3_L + H	–6.88 (2)	-	-	-	-	-
3Cu+2L ⇌ Cu_3_H_–2_L_2_ + 2H	17.80 (3)	-	-	-	-	-
Cu_3_H_-2_L_2_ ⇌ Cu_3_H_–3_L_2_ + H	-8.14 (9)	-	-	-	-	-
pCu^c^	9.8	13.8	9.9	10.6	11.5	9.8

a Charges omitted.

b Numbers in parentheses are standard deviations in the last significant figure.

c pCu values calculated for [L] = 1 × 10^–5^ M and [Cu^2+^] = 1 × 10^–6^ M.

This low number of coordinated nitrogen atoms is supported by a couple of crystal structures we have obtained for Cu^2+^ complexes of **L1**. The first crystals (**1**) grew when slowly evaporating an aqueous solution of **L1**·4HBr and Cu­(ClO_4_)_2_·6H_2_O at pH ca. 6. The crystals of formula [Cu­(H**L1**)­Br_2_]­Br·H_2_O consisted of [Cu­(H**L1**)­Br_2_]^+^ cations, Br^–^ counter-anions, and bulk water molecules. The metal is coordinated by the tertiary and one of the secondary amine groups of the macrocyclic core and the primary amine group of the tail. The square pyramidal coordination (Addison et al. parameter, λ = 0.11)^31^ is completed by two bromide anions, one of them occupying the distorted axial position (Figure 10). The elevation of the copper ion over the equatorial plane is just 0.123 Å. Table 3 shows selected bond distances and angles. The noncoordinated secondary amine group of the macrocyclic core is protonated in the crystal.

**Figure 10:**
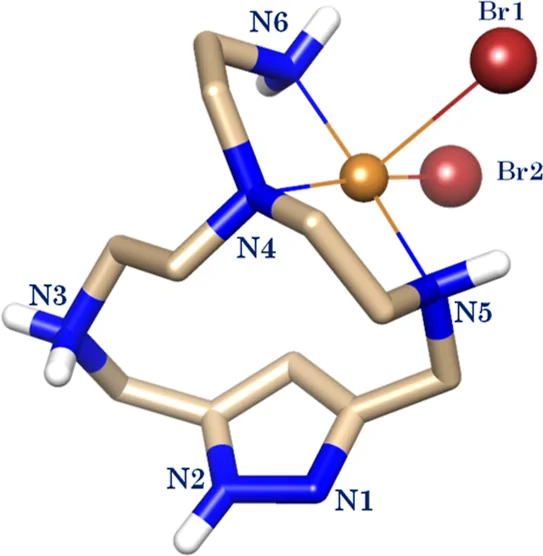
Ball and stick view of the complex cation [Cu­(H**L1**)­Br_2_]^+^.

**Table 3:** Selected Bond Distances (Å) and Angles (^0^) for **1**

bond distances (Å)		angles (^0^)	
Cu–N4	2.082(3)	N4–Cu–N5	86.5(1)
Cu–N5	2.030(3)	N4–Cu–N6	85.2(1)
Cu–N6	1.988(3)	N5–Cu–Br1	98.73(9)
Cu1–Br1	2.4167(5)	N6–Cu–Br1	90.2(1)
Cu1–Br2	2.8824(6)	Br2–Cu–N4	101.75(9)
		Br2–Cu–N5	83.44(9)
		Br2–Cu–N6	94.5(2)
		Br2–Cu1–Br1	93.73(2)

Interestingly enough, the nitrogen atoms of the pyrazole ring are not coordinating the metal ion, which may be valuable for assisting catalytic applications.

In the crystal structure, the [Cu­(H**L1**)­Br_2_]^+^ cations are forming chains interconnected through a hydrogen bond network involving the protonated secondary amine group of the macrocycle (N3) and the bromide anion at the apical position (Br2) of the Cu^2+^ coordination sphere, and between the equatorial bromides (Br1) and the water molecules (O3) and the protonated pyrazole nitrogen N2. The protonated amine is forming further hydrogen bonds with bromide counter-anions (Br3). The different chains are in turn interconnected by π-stacking between the pyrazole rings, whose average planes are at a distance of 3.26 Å, while the distance between centroids is 4.168 Å (Figure S10).

The second crystals (**2**) have a quite different structure and were obtained when evaporating aqueous solutions of **L1**·4HBr and Cu­(ClO_4_)_2_·6H_2_O at pH ca. 3. At this more acidic pH, the distribution diagram calculated at millimolar concentrations do not show complex formation; however, an increase of the concentration due to evaporation leads to the formation of crystals of formula [Cu_2_(H_3_LBr)_2_Br_6_]­(ClO_4_)_4_·4H_2_O (**2**). These crystals present particular features that deserve analysis. First, the primary and secondary amines of both macrocycles are fully protonated and, therefore, cannot coordinate the metal ions, which are coordinated by the pyrazole spacers in a monodentate fashion. Each copper ion completes its square pyramidal coordination geometry with four bromide anions; those placed in the axial position behave as a double μ-bridge between the metals, the Cu–Cu distance is 3.725(1) Å, and the Cu–Br–Cu angle is close to 90° (Figure 11 and Table 4). Interestingly, the CH group of both pyrazole spacers is brominated, a feature that was not observed in the absence of the coordinated metal.

**Figure 11:**
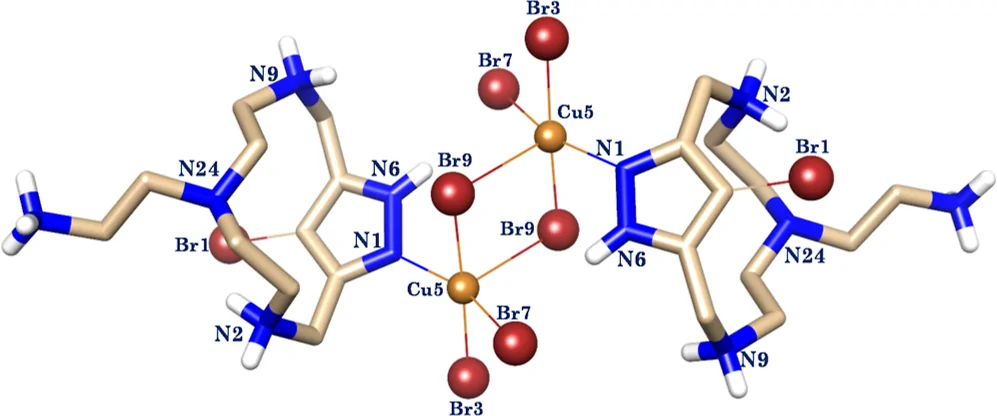
View of the [Cu_2_(H_3_ **L1**Br) _2_Br_6_]^4+^ binuclear complex unit.

**Table 4:** Selected Bond Distances (Å) and Angles (^0^) for **2**

bond distances (Å)		angles (^0^)	
Cu–Br3	2.418(1)	N1–Cu–Br9	87.6(2)
Cu–Br7	2.391(1)	N1–Cu–Br3	91.0(2)
Cu–N1	2.035(7)	Br3–Cu–Br7	91.54(5)
Cu–Br9	2.792(1)	Br7–Cu–Br9	89.16(2)
Cu–Cu	3.725(1)	Br9–Cu–Br9	88.02(4)
		Br9–Cu–Br7	103.38(5)
		Br9–Cu–Br3	93.88(4)
		Br9–Cu–Nr1	97.8(2)
		Cu–Br9–Cu	91.98(2)

The binuclear units are interconnected through a wide hydrogen-bonding framework in which participate the two secondary ammonium groups of the macrocyclic, the primary ammonium group of the tail, and the noncoordinated N–H of pyrazole.

The crystal structure of **1** explains the low stability of these complexes and also why, in the case of pztren, monomeric and dimeric complexes with higher nuclearity were found since the amine groups of the chain and the pyrazole unit do not participate in the metal coordination of the mononuclear complexes. Interestingly, complexes with analogous stoichiometries were found in the case of copper complexes of condensed pyrazole azacyclophane ligands [1 + 1],^16^ whose formation was regulated by the length and number of nitrogen atoms in the polyamine bridge.

All these studies allow us to conclude that these macrocycles do not behave as proper scorpiand ligands since the donor atoms of the pendant arm are always involved in coordination to the metal, while the pyrazole spacer and one of the secondary amine groups of the macrocyclic core do not appear to participate in the binding. Therefore, the motion of the pendant arm is prevented since the metal ion would not exclusively reside in the macrocyclic core. Nevertheless, the low coordination number achieved by the metal ion and the uneven binding mode can make these systems appropriate for developing catalytic systems, as, for instance, in mimicking antioxidant enzymes. In the next section, we will make a preliminary analysis of the potential of these systems to behave as SOD mimics.

### Electrochemistry

A key point for a metal complex to be able to act as an SOD mimic is that its redox potential at physiological pH must be less negative than the reduction potential for the oxygen/superoxide anion couple (*E* ^0^ O_2_/O_2_^.‑^ = −0.33 V vs normal hydrogen electrode (NHE)) but less positive than the superoxide anion/water couple (*E* ^0^ O_2_^.‑^/H_2_O_2_ = +0.89 V vs NHE).^32^

The studied systems display a common voltammetric pattern, exemplified in Figure 10 for the system Cu^2+^-**L1,** where initial cathodic scan cyclic voltammograms reversed at different potentials are depicted. In the extended potential range, two cathodic peaks at −0.22 (C1) and −0.50 V (C2) vs Ag/AgCl appear preceding the rising current at ca. −1.0 V corresponding to the hydrogen evolution reaction (HER). In the reverse scan, a tall anodic peak appears at −0.10 V (A2) preceding a wave at +0.20 V (A1). Upon switching the potential to −0.30 V, the cathodic wave C1 does not show any significant anodic counterpart, while the prominent peak A2 disappears (Figure 12). To rationalize this voltammetric behavior, two features must be considered: (i) the peak A2 can unambiguously be attributed to the oxidative dissolution (stripping) of a deposit of metallic copper formed in the previous reductive steps; (ii) the solution contains halide counterions.

**Figure 12:**
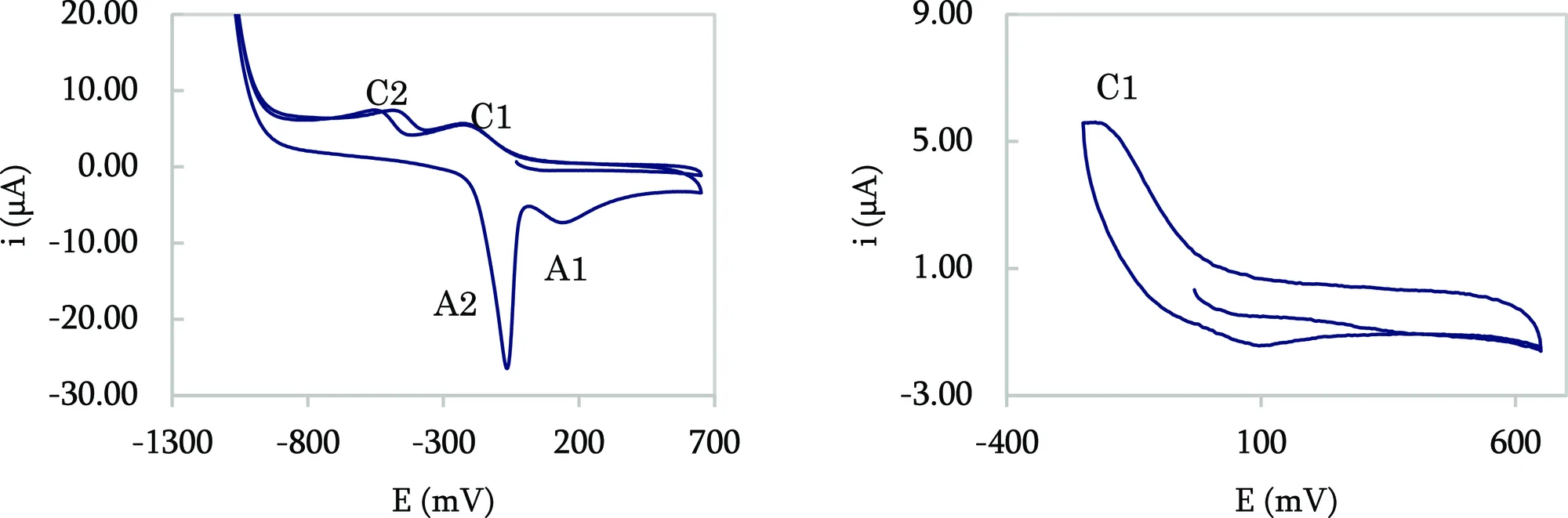
Cyclic voltammograms of a deoxygenated 0.5 mM **L1** plus 0.4 mM Cu­(II) solution in 0.10 M Tris at pH 7.4. Potential scan reversed at A: −1.25 V and B: −0.30 V vs Ag/AgCl. Potential scan rate 50 mV s^–1^.

Therefore, this electrochemical behavior can be interpreted assuming that the parent complex Cu^2+^-L undergoes an initial, apparently irreversible, one-electron reduction (process C1). The resulting Cu^+^-L species experiences a more or less slow reorganization reaction, yielding a Cu^+^ species that is reduced to Cu metal through process C2, with complex disintegration. The oxidation of the metallic deposit produces the stripping peak A2, presumably releasing Cu^+^ ions that are complexed with halide ions more rapidly than the receptors. The resulting Cu^+^-halide complexes are oxidized in step A1 to the corresponding Cu^+^-halide ones, which rapidly re-equilibrate with the excess of ligand, yielding the parent Cu^2+^-L.

For our purposes, the relevant point to emphasize is that the apparent irreversibility of the process C1 does not allow us to calculate the redox potential of the Cu^2+^-L/Cu^+^-L couple. A reasonable estimate can be made assuming that the formal potential of the couple can be approximated by the half-peak potential (*E*_p/2_) of the process C1. Table 5 summarizes the formal potentials (*E*°’) evaluated for the different complexes and referred to the NHE. In all cases, *E*°’ falls within the interval defined by the standard potentials of the O_2_/O_2_^•‑^ and O_2_^.‑^/H_2_O_2_ couples, which is the thermochemical condition for SOD activity to occur.

**Table 5:** Formal Electrode Potentials Estimated for the Cu^2+^-L/Cu^+^-L Couple for the Complexes in This Study

system	*E*°’ (V vs NHE)	reaction
O_2_/O_2_^•‑^	–0.33	O_2_ + e^–^ → O_2_^•‑^
O_2_^•‑^/H_2_O_2_	0.89	O_2_^•‑^ + 2H^+^ + e^–^ → H_2_O_2_
Cu-L1	0.15	[Cu(II)L1] + e^–^ → [Cu(I)L1]
Cu-L2	0.06	[Cu(II)L2] + e^–^ → [Cu(I)L2]
Cu-L3	0.15	[Cu(II)L3] + e^–^ → [Cu(I)L3]
Cu-L4	0.20	[Cu(II)L4] + e^–^ → [Cu(I)L4]
Cu-L5	0.08	[Cu(II)L5] + e^–^ → [Cu(I)L5]
Cu-L6A	0.07	[Cu(II)L6A] + e^–^ → [Cu(I)L6A]
Cu-L6B	–0.08	[Cu(II)L6B] + e^–^ → [Cu(I)L6B]

The voltammetric response of all the other complexes studied was similar. The general pattern consists of two more or less overlapped cathodic peaks around −0.22 and (C1) and −0.50 V (C2) vs Ag/AgCl. During the subsequent anodic sweep, a characteristic stripping peak was observed at −0.10 V (A2), followed by a second, less intense oxidation feature appearing between −0.15 and +0.08 V (A1), whose position depended on the complex under investigation. Studies carried out over different potential windows indicated that the A2 process is directly associated with the preceding cathodic signal C2, suggesting that this reduction step results in the formation of metallic copper. By contrast, the C1 signal is assigned to the reduction of Cu^2+^ to Cu^+^. This process is typically detected either as a distinct peak (**L1**, **L3**) or as a shoulder (**L2**, **L6**) located before the main cathodic peak C2. It should be noted that in the case of **L5,** the cyclic voltammogram displays two Cu^2+^ to Cu^+^ reductions (C1 and C2) and, with wider potential windows, two additional reduction waves (C3 and C4) associated with the formation of Cu^0^. The well-defined anodic peak corresponds to the oxidation of metallic copper. Analysis at different scan rates suggests the presence of two distinct Cu^+^ complexes, generated through structural reorganization, and two formal potentials are obtained.

### Evaluation of the SOD Activity

Taking into account the promising SOD activity of the pyridine analogue compounds,^11^ the unfilled coordination spheres shown by these scorpiand complexes, and the ability of the uncoordinated pyrazole heteroatoms to donate and accept hydrogen bonds, we performed a preliminary assay to evaluate the capacity of the Cu^2+^ complexes to scavenge superoxide radical anions by using the McCord–Fridovich indirect method,^33^ that allows to calculate a IC_50_ and catalytic constant a physiological pH. As shown by the IC_50_ values in Table 6 and Figure S11, all the Cu^2+^ complexes, except Cu^2+^-**L2**, exhibit a remarkable antioxidant activity; the IC_50_ values are just slightly over 1 order of magnitude less active than the native enzyme. The *k*_cat_ values obtained indicate that these systems rank among the potentially best SOD mimics reported to date.^34–37^ The IC_50_ values of these complexes are slightly higher than those we have recently reported for the binuclear complexes of related hexaza-pyrazolacyclophanes. Interestingly, the less active system is the pyridine substituted at the 2-position, which is the one showing the highest stability (Table 2). This behavior can be attributed to the fact that this system possesses a coordination number and geometry better suited to accommodate Cu^2+^ and, therefore, shows a lower tendency to be reduced to Cu^+^, a step that is essential in the characteristic ping-pong cycling mechanism of SOD.^38,39^

**Table 6:** IC_50_ and *K*_cat_ Values of the Cu­(II)-**L1**, Cu­(II)-**L2**, Cu­(II)-**L3**, Cu­(II)-**L4**, and Cu­(II)-**L6** Complexes, Compared with Those of the Native CuZn-SOD Enzyme and Cu­(ClO_4_)_2_

system	IC_50_ (μM)	*k*_cat_ (10^6^ M^–1^s^–1^)	reference
Cu(II)-**L1**	0.35 (2)	10.6	-
Cu(II)-**L2**	1.00 (3)	3.6	-
Cu(II)-**L3**	0.45 (5)	8.3	-
Cu(II)-**L4**	0.40 (2)	8.1	-
Cu(II)-**L6**	0.30 (3)	12.4	-
CuZn-SOD	0.010 (2)	430.0	^40,41^
Cu(ClO_4_)_2_	1.1 (1)	2.7	^39,40^

Further studies are currently being done to assess other aspects of this antioxidant behavior and their role in in vitro and in vivo experiments.

## Conclusions

Starting from a macrocycle constituted by a *tris*(2-aminoethyl)­amine linked through methylene groups to the 3 and 5-positions of an 1*H*-pyrazole spacer, we have prepared with reasonable yields a family of ligands bearing either pyridine or quinoline aryl groups connected through a methylene bridge to the amine group of the pendant arm. Potentiometric studies, UV–vis spectra, and NMR data indicate that the nonmetalated ligands undergo conformational changes in which the pendant arm moves toward or away from the macrocyclic core, depending on the protonation degree of the compounds. However, coordination of the Cu^2+^ to form complexes of 1:1 stoichiometry blocks the bending movements due to the simultaneous involvement of donor atoms of both the pendant arm and the macrocyclic core in metal binding. This behavior is supported by the analysis of the Cu^2+^ complex stability constants and by spectroscopic data. The crystal structure of the compound [Cu­(H**L1**)­Br_2_]­Br·H_2_O confirmed this interpretation and reveals the presence of non-coordinated donor atoms within the macrocyclic core. All these features prompted the idea that these complexes may behave as potential candidates for mimicking enzymes involved in protection against reactive oxygen species. Preliminary analysis showed excellent potential to behave as SOD mimics, ranking among most the best candidates reported to date.

## Experimental Section

The synthesis of **L1-L6** was performed, and the resulting compounds gave satisfactory elemental microanalysis and spectroscopic characterization. 1*H*-3,5-*Bis*(chloromethyl)­pyrazole and 3,5-bis­(chloromethyl)-1-(tetrahydropyran-2-yl)-pyrazole were prepared according to previously reported methods,^42,43^ and the tosylated amine was synthesized following the procedure previously reported.^9^ ^a^

### 6-(2-Aminoethyl)-3,6,9-triaza-1-(3,5)-pyrazolacyclodecaphane

The tosylated polyamine tren (5.0 mmol) and K_2_CO_3_ (50.0 mmol) were suspended in 250 mL of anhydrous acetonitrile in a round-bottom flask. 3,5-Bis­(chloromethyl)-1-(tetrahydropyrane-2-yl)-pyrazole (5.0 mmol) was dissolved in 150 mL of anhydrous acetonitrile and dropwise added over 1 h. The suspension was refluxed for 48 h under a nitrogen atmosphere and then filtered. The solution was vacuum-evaporated to dryness. Purification was carried out by column chromatography (silica gel, dichloromethane/acetone 25/1) to give the product as a white solid. Yield: 64%. ^1^H NMR (300 MHz, CDCl_3_): δ (ppm) 7.81–7.59 (m, 6H), 7.40–7.27 (m, 6H), 6.47 (s, 1H), 5.65–5.49 (m, 1H), 5.17–5.05 (m, 1H), 4.09–3.95 (m, 1H), 3.88–3.71 (m, 1H), 2.95–2.83 (m, 2H), 2.45 (s, 3H), 2.43 (s, 3H), 2.38 (s, 3H), 2.35–3.27 (m, 2H), 2.08–1.84 (m, 2H), 1.80–1.46 (m, 3H). ^13^C NMR (75 MHz, CDCl_3_): δ (ppm) 147.3, 144.1, 138.3, 137.0, 135.2, 133.7, 130.3, 127.6, 111.1, 84.3, 76.7, 68.2, 52.8, 50.3, 48.5, 45.6, 44.7, 42.3, 40.4, 31.0, 24.9, 22.7, 21.7.

### Synthesis of 6-(2-aminoethyl)-3,6,9-triaza-1-(3,5)- Pyrazolacyclodecaphane Tetrahydrobromide (L1·4HBr)

The protected macrocycle (2.0 mmol) and phenol (12 equiv per protective group) were suspended in 33% HBr-AcOH (12 L per protective group). The mixture was stirred at 90 °C for 24 h and then cooled. The resulting residue was filtered and washed with acetone to give L1 as its hydrobromide salt. Yield: 43%. ^1^H NMR (300 MHz, D_2_O) (ppm) 7.05 (s, 1H), 4.46 (s, 2H), 3.23–2.98 (m, 3H), 2.68 (t, *J* = 6.8 Hz, 1H). ^13^C NMR (75 MHz, D_2_O): δ (ppm) 139.96, 110.15, 50.24, 47.81, 42.83, 42.04, 35.59. ESI-MS (*m*/*z*): Calculated for [L + H]^+^: 239.2. Found: 239.1. Elemental analysis: Calculated for C_11_H_22_N_6_·4HCl·2H_2_O·C_2_H_6_O: C (32.5%); H (7.4%); N (19.3%). Found: C_11_H_22_N_6_·4HCl·2H_2_O·C_2_H_6_O: C (32.9%); H (6.8%); N (19.2%).

For the functionalization of ligands L2-L6, a prior deprotection step is required to obtain ligand L1 in its free amine form, since after removal of the protecting groups, it is obtained as the hydrobromide salt. This conversion is achieved using an anion-exchange resin (Amberlite IRA-402). The final reaction is carried out by dissolving L1 (3.0 mmol) in anhydrous ethanol and adding dropwise an equimolar solution of the corresponding carboxaldehyde, while stirring under an inert atmosphere at room temperature for 2 h. Subsequently, NaBH_4_ is added, and the reaction mixture is stirred overnight. After solvent evaporation and extraction with dichloromethane, the product is purified and precipitated as the hydrochloride salt by addition of 4 M HCl in 1,4-dioxane. In some cases, anhydrous methanol was used instead of ethanol, and traces of this solvent were detected in the elemental analysis.

### Synthesis of 6-[4-(2-pyridil)-3-azabutyl]-3,6,9-triaza-1-(3,5)-pyrazolacyclodecaphane Tetrahydrochloride (L2·4HCl)

Yield: 25%. ^1^H NMR (300 MHz, D_2_O): δ (ppm) 8.89 (d, *J* = 6.3 Hz, 2H), 8.18 (d, *J* = 6.1 Hz, 2H), 7.04 (s, 1H), 4.61 (s, 2H), 4.43 (s, 4H), 3.32 (d, *J* = 5.3 Hz, 2H), 3.11 (q, *J* = 6.5 Hz, 4H), 2.80 (t, *J* = 7.1 Hz, 2H). ^13^C NMR (75 MHz, D_2_O): δ (ppm) 147.79, 147.03, 142.03, 139.92, 125.76, 110.20, 49.79, 48.83, 43.80, 42.83, 42.00. ESI-MS (*m*/*z*): Calculated for [L + H]^+^: 330.2401. Found: 330.2808. Elemental analysis: Calculated for C_17_H_27_N_7_·4HCl·4H_2_O·CH_3_OH: C (37.6%); H (6.8%); N (17.04%). Found: C_17_H_27_N_7_·4HCl·4H_2_O·CH_3_OH: C (37.6%); H (7.1%); N (17.3%).

### Synthesis of 6-[4-(3-Pyridil)-3-azabutyl]-3,6,9-triaza-1-(3,5)-pyrazolacyclodecaphane Tetrahydrochloride (L3·4HCl)

Yield: 53%. ^1^H NMR (300 MHz, D_2_O): δ (ppm) 9.02 (m, 1H), 8.92 (dd, *J* = 6.6, 1.5 Hz, 1H), 8.82–8.75 (m, 1H), 8.19 (dd, *J* = 8.2, 5.8 Hz, 1H), 7.05 (s, 1H), 4.56 (s, 2H), 4.45 (s, 4H), 3.30 (t, *J* = 7.0 Hz, 2H), 3.13 (t, *J* = 7.0 Hz, 4H), 2.85–2.74 (t, *J* = 7.0 Hz, 2H). ^13^C NMR (75 MHz, D_2_O): δ (ppm) 148.30, 142.76, 142.66, 139.93, 130.99, 127.85, 110.19, 49.06, 47.75, 47.62, 43.97, 42.82, 42.02. ESI-MS (*m*/*z*): Calculated for [L + H]^+^: 330.2401. Found: 330.2410. Elemental analysis: Calculated for C_17_H_27_N_7_·4.5HCl·4H_2_O·CH_3_OH: C (36.3%); H (7.4%); N (16.5%). Found: C_17_H_27_N_7_·4.5HCl·4H_2_O·CH_3_OH: C (36.6%); H (6.0%); N (16.1%).

### Synthesis of 6-[4-(4-Pyridil)-3-azabutil]-3,6,9-triaza-1-(3,5)-pyrazolacyclodecaphane Tetrahydrochloride (L4·4HCl)

Yield: 38%. ^1^H NMR (D_2_O, 300 MHz): δ (ppm) 8.8 (d, J = 8.8, 2H), 8.1 (d, J = 8.1, 2H), 7.0 (s, 1H), 4.59 (s, 2H), 4.40 (s, 4H), 3.3–3.2 (m, 2H), 3.1–3.0 (m, 4H), 2.78–2.69 (m, 2H). ^13^C NMR (D_2_O, 75 MHz): δ (ppm) 139.87, 137.71, 125.31, 107.99, 47.41, 46.71, 45.51, 42.06, 40.60, 39.81. ESI-MS (*m*/*z*): Calculated for [L + H]^+^: 330.2406. Found: 330.2412. Elemental analysis: Calculated for C_17_H_27_N_7_·4HCl·6H_2_O: C (35.2%); H (6.8%); N (16.9%). Found: C_17_H_27_N_7_·4HCl·6H_2_O: C (35.3%); H (6.7%); N (16.6%).

### Synthesis of 6-[4-(2-quinolil)-3-azabutil]-3,6,9-triaza-1-(3,5)-pyrazolacyclodecaphane Tetrahydrochloride (L5·4HCl)

Yield: 30%. ^1^H NMR (300 MHz, D_2_O): δ (ppm) 2.83 (t, *J* = 6.9 Hz, 2H), 3.12 (t, *J* = 6.7 Hz, 4H), 3.34 (t, *J* = 6.9 Hz, 2H), 4.44 (s, 4H), 4.65 (s, 2H), 7.04 (s, 1H), 7.73­(d, *J* = 1.2 Hz, 1H), 7.77–7.82 (m, 1H), 7.94–8.00­(m, 1H), 8.14 (t, *J* = 9.0 Hz, 2H), 8.65 (d, *J* = 9.7 Hz, 1H). ^13^C NMR (75 MHz, D_2_O): δ (ppm) 148.69, 144.35, 141.94, 139.90, 133.96, 129.48, 128.82, 128.45, 123.56, 121.31, 110.26, 49.46, 49.11, 47.76, 44.26, 42.81, 42.00. ESI-MS (*m*/*z*): Calculated for [L + D]^+^: 381.2641. Found: 381.2605. Elemental analysis: Calculated for C_21_H_29_N_7_·5HCl·4H2O: C (39.92%), N (15.53%), H (6.70%). Found: C_21_H_29_N_7_·5HCl·4H_2_O: C (40.10%), N (15.300%), H (6.10%).

### Synthesis of 6-[4-(4-quinolil)-3-azabutil]-3,6,9-triaza-1-(3,5)-pyrazolacyclodecaphane Tetrahydrochloride (L6·4HCl)

Yield: 54%. ^1^H NMR (300 MHz, D_2_O): δ (ppm) 9.22 (d, *J* = 5.7 Hz, 1H), 8.45 (d, *J* = 8.5 Hz, 1H), 8.34 (d, *J* = 8.7 Hz, 1H), 8.29–8.18 (m, 2H), 8.10 (m, 1H), 7.08 (s, 1H), 5.14 (s, 2H), 4.46 (s, 4H), 3.47 (t, *J* = 7.2 Hz, 2H), 3.19–3.04 (m, 7H), 2.89 (t, *J* = 7.3 Hz, 2H), 2.68 (t, *J* = 6.8 Hz, 1H). ^13^C NMR (75 MHz, D_2_O): δ (ppm) 148.54, 144.08, 139.96, 137.75, 135.42, 131.05, 126.83, 124.23, 121.63, 121.08, 110.26, 48.88, 47.76, 47.19, 42.84, 42.07. ESI-MS (*m*/*z*): Calculated for [L + D]^+^: 381.2635. Found: 381.2605. Elemental analysis: Calculated for C_21_H_29_N_7_·5HCl·4H_2_O: C (39.9%); H (6.7%); N (15.5%). Found: C_21_H_29_N_7_·5HCl·4H_2_O: C (39.9%); H (5.7%); N (15.5%).

^1^H NMR and ^13^C NMR spectra are provided in Figures S12 and S23. Theoretical and calculated mass spectra are collected in Figures S24–S29.

#### Synthesis of [Cu­(HL1)­Br_2_]­Br·H_2_O (1)

Crystals of 1 were grown by slow evaporation of an aqueous solution of L1·4HBr and Cu­(ClO_4_)_2_·6H_2_O at pH ca. 6 in an open vessel. After several days, crystals of 1 suitable for single X-ray crystal diffraction were obtained. Anal. Calcd for C_11_H_25_Br_3_N_6_CuO: C, 23.57; H, 4.50; N, 15.00. Found: C, 23.3; H, 4.7; N, 15.1. Cambridge Crystallographic Data Centre (CCDC) 2531565.

#### Synthesis of [Cu_2_(H_3_BrL1)_2_Br_6_]­(ClO_4_)_4_·4H_2_O (2)

Crystals of 2 were grown by slow evaporation of an aqueous solution of L1·4HBr and Cu­(ClO_4_)_2_·6H_2_O at pH ca.3 in an open vessel. After several days crystals of 2 suitable for single X-ray crystal diffraction were obtained. Anal. Calcd for C_22_H_56_Br_8_N_12_Cu_2_Cl_4_O_20_: C, 15.35; H, 3.28; N, 9.76. Found: C, 15.2; H, 3.2; N, 10.0. CCDC 2531564.

Perchlorate salts of compounds containing organic fragments can be explosive and should be handled with care.

### Electromotive Force Measurements

The potentiometric titrations were carried out at 298.1 ± 0.1 K using NaCl 0.15 M as supporting electrolyte. The experimental procedure (buret, potentiometer, cell, stirrer, microcomputer, etc.) has been fully described elsewhere.^44^ The acquisition of the *emf* data was performed with the computer program PASAT.^45^ The reference electrode was a Crison 52 40 Ag/AgCl electrode in 0.5 M NaCl solution. A Wilhelm bridge filled with 0.5 M NaCl was used to separate the glass and the reference electrode. The glass electrode (Crison 52 50 Ag/AgCl) was calibrated as a hydrogen-ion concentration probe by titration of previously standardized amounts of HCl with CO_2_-free NaOH solutions and determining the equivalent point by Gran’s method,^46,47^ which gives the standard potential, *E*°’, and the ionic product of water (pKw = 13.73(1)). The computer program HYPERQUAD^20^ was used to fit the protonation and stability constants. Solutions containing the ligand salts with Cu^2+^:L molar ratios varying from 2:1, 3:2, and 1:2 were titrated with NaOH with Cu^2+^ concentrations ranging from 2.0 × 10^–3^ M, 1.5 × 10^–3^ M, and 1.1 × 10^–3^ M. The different titration curves for each system were treated as separate curves without significant variations in the values of the stability constants. Finally, the sets of data were merged together and treated simultaneously to give the final stability constants. When more than one model fit the experimental data, the most reliable chemical model was chosen by performing F tests at the 0.05 confidence level.^48,49^

### NMR Measurements

The ^1^H and ^13^C NMR spectra were recorded on Bruker Advance DPX 300 MHz and Bruker Advance DPX 400 MHz spectrometers operating at 299.95 and 399.95 MHz for ^1^H and at 75.43 and 100.58 MHz for ^13^C^50^ and on Bruker Advance Neo500 (with a 500 MHz, 11.7 T Bruker magnet) from SCSIE at the University of Valencia operating at 500.13 MHz for ^1^H and at 125.77 MHz for ^13^C. Deuterated solvents were used to perform the NMR experiments. Sodium 3-(trimethylsilyl)­propionate-2,2,3,3-d_4_ (TSP-d_4_) was used as a standard reference in the NMR studies in D_2_O at d 0.00 ppm. To adjust the desired pD, DCl and NaOD solutions were used. The pD was calculated from the measured pH values using the correlation, pH = pD-0.4.^51^ The 2D HSQC phase-sensitive ge-2D ^1^H–^13^C spectrum was collected with Bruker Neo500 with BBOF Plus ATM with a spectral width of 12 ppm in F2 (1H) dimension and 239 ppm in the F1 (^13^C) dimension. A total of 1024 points were collected in F2 with 128 increments in F1 by using 80 scans per increment. The 1 JCH was set to 145 Hz. 2D^1^ H–^1^H COSY spectra were acquired with Neo500 with BBOF Plus ATM at 298 K in D_2_O. The COSY pulse sequence was employed, incorporating a 1.5 s relaxation delay with presaturation of the residual water resonance at d 4.70 ppm. A total of 128 increments in t1 were collected with 32 scans per increment and spectral width of 12 ppm in both dimensions. The solvent suppression was achieved via a low-power presaturation pulse, which was optimized to 40.8 dB.

### Computational Studies

Molecular dynamics simulations were carried out using Amber 2019, explicit solvent (10 Å TIP3P water box and Cl^–^ as counterion), and GAFF potentials at 300 K.^52^ The process comprised different stages: solvation, minimization, and a heating/equilibration stage (in several steps) until a final temperature of 300 K was reached, after which a 3 ns simulation was done. The compounds were modeled using the GAFF potentials, and the environmental conditions were simulated using the ionsjc_tip3p field. No restraint at all was used in these studies. Topology and trajectory files in AMBER format are available on request.

### X-Ray Analysis

The crystals were measured in a Bruker D8 Venture X-ray diffractometer using Mo Kα radiation (λ = 0.71073Å) equipped with an Oxford low temperature unit operating at 120 K. Indexing, strategy, and data collection were performed with APEX3 software suite. OLEX2 ^53^ was used as a frontend for solving and refining. Initial structure was solved with direct methods using SHELXS and then refined with SHELXL2018.^54^ Initially, an isotropic refinement was performed on the non-hydrogen atoms. Then, anisotropic refinement was done.

### Mass Spectrometry

HR-ESI mass spectra of solutions (water/methanol 50/50 vol/vol) containing a given ligand and Cu­(ClO_4_)_2_·6H_2_O in 1:1, 2:1, and 2:3 molar ratio were acquired in the positive ion mode using a Triple TOF 5600 hybrid quadrupole time-of-flight (TOF) mass spectrometer. N_2_ was used as a curtain and nebulizing gas. The experiments were performed at a voltage of 5300 V and GS1 and GS2 (35 psi) ion source gas at 723.15 K. The AB SCIEX Peak View software was used for the analysis of the data.

### UV–vis Spectroscopy

UV–vis spectra of the ligand and its complexes at a 1:1 molar ratio were recorded at 298.15 K using an Agilent 8453 UV–vis spectrophotometer. All spectroscopic measurements were performed in 0.15 M NaCl solutions, and the corresponding NaCl solution was used as a baseline reference. Spectral data below 200 nm were excluded from the analysis because of the significant absorbance of chloride ions in this wavelength region.

### Electrochemical Measurements

Cyclic voltammetry experiments were performed on Cu­(ClO_4_)_2_·6H_2_O plus ligand solutions in 0.10 M Tris buffer at pH 7.4. Electrochemical experiments were performed with a CH 440I potentiostat in a conventional three-electrode cell using a glassy-carbon working electrode (BS MF2012, geometrical area 0.071 cm^2^) previously cleaned and activated with an aqueous suspension of alumina on a soft surface, dried, and cleaned. An AgCl (3 M NaCl)/Ag and a platinum-wire auxiliary electrode completed the three-electrode arrangement. The cyclic voltammograms were recorded at scan rates of 10–2000 mV s^–1^ in solutions optionally deaerated by bubbling Ar for 10–15 min.

### SOD Assay

The SOD-like activity was determined by using the nitro blue tetrazolium (NBT) method.^55^ The assays were carried out in a pH = 7.4 50 mM HEPES buffer at 298.1 K. The xanthine (2.2 × 10^–4^ M)/xanthine oxidase system was used to generate a reproducible and constant flux of superoxide anions. The reduction rate of NBT (7.3 × 10^–5^ M) to blue formazan was followed spectrophotometrically at 560 nm. Data in the absence of the complex were used as a reference. The rate of NBT reduction was inhibited after the addition of the complex solutions at increasing concentrations prepared in 50 mM Tris–HCl buffer. The percentage of inhibition of the NBT reduction was used as a measure of the SOD activity of the compounds. The concentration of complex required to yield 50% inhibition of NBT reduction (IC_50_) was determined from a plot of percentage inhibition versus complex concentration. The IC_50_ data have been calculated from the mean values of at least three independent measurements. The catalytic constant was calculated from the IC_50_ using the equation *k*_cat_ = *k*_NBT_[NBT]/IC_50_, where *k*_NBT_= (5.9 ± 0.5) × 10^5^ M^–1^ s^–1^.^56^ Blank experiments were recorded with the ligands without observing any effect.

## Supplementary Material





## Notes

Distribution diagrams of the ligands (S1, S2, S5, and S7) and complexes, variation of the chemical shifts of **L1** in the pD range 2.16–6.41, temperature dependence of the ^1^H NMR spectrum of **L1**, variation with pD of the ^13^C NMR spectra of **L3**, plot of pCu vs pH, details of the three-dimensional arrangement of [Cu­(H**L1**)­Br_2_]^+^, ^1^H and ^13^C NMR spectra of the synthesized ligands, HR-ESI mass spectra of the ligands, and table of crystallographic data for (**1**) and (**2**) (PDF)
Videos illustrating the molecular dynamics simulations (ZIP)
