# Supplementary material for: Scorpiand Pyrazole Azacyclophanes: Synthesis, Protonation Behavior, and Cu2+ Coordination Chemistry
Source: Inorg Chem. 2026 Aug 27;65(36):20898–912. doi: 10.1021/acs.inorgchem.6c02243 (PMC13580195; doi:10.1021/acs.inorgchem.6c02243)
Supplement: Supplementary file 1 [file ic6c02243_si_001.pdf]

Supporting Information

Scorpiand Pyrazole Azacyclophanes: synthesis, protonation behavior and Cu^{2+} Coordination Chemistry

Irene Bonastre-Sabater,[a] Alberto Lopera,[a] Salvador Blasco,[a] José M. Llinares,[b] Mario Inclán,[c] Antonio Doménech-Carbó,[d] Enrique García-España,[a] and M. Paz Clares.[a,b,*].*

a) Departamento de Química Inorgánica, Instituto de Ciencia Molecular, Universidad de Valencia, C/Catedrático José Beltrán 2, 46980 Paterna (Valencia), Spain. E-mail: enrique.garcia-es@uv.es

b) Departamento de Química Orgánica, Facultad de Farmacia y Ciencias de la Alimentación, Universidad de Valencia, Av. Vicent Andrés Estellés, n° 22. 46100. Burjassot (Valencia). Spain E-mail: m.paz.clares@uv.es

c) Departamento de Farmacia, Facultad de Ciencias de la Salud, Universidad CEU Cardenal Herrera, Lluís Vives, 1, 46115 Alfara del Patriarca, (Valencia), Spain.

d) Departamento de Química Analítica, Facultad de Química, Universitat de València, Av. Vicent Andrés Estellés, 19C. 46100. Burjassot (Valencia).

Contents

I. Figures

Figure S1. Distribution diagrams for **L1**-**L6** in the pH range 2.5-11.0.

Figure S2. Variation with the pH of the UV spectra of **L1** (left) and variation of the absorbance at 203 nm overlapped with the distribution diagram (right).

Figure S3. Variation of the chemical shifts of **L1** in the pD 2.16-6.41.

Figure S4. ^{1}H NMR spectra of **L1** at 298 K (top) and 353 K (bottom).

Figure S5. A) Variation of the UV-Vis spectra of **L3** with pH. B) Variation of the band centered at 210 nm with pH overlapped with the distribution diagram. [L] = 1×10^{-4} M.

Figure S6. Variation of the ^{13}C NMR spectra of **L3** with pH.

Figure S7. A) Variation of the UV-Vis spectra of **L6** with pH. B) Variation of the band centered at 219 nm with pH overlapped with the distribution diagram. [L] = 1×10^{-4} M.

Figure S8. Distribution diagrams of the systems A) Cu(II)-**L1**, B) Cu(II)-**L2**, C) Cu(II)-**L3**, D) Cu(II)-**L4,** E) Cu(II)-**L5** and F) Cu(II)-**L6** related to Cu(II) in 1:1 molar ratio calculated from the potentiometric constants. In NaCl 0.15 M. [L] = [Cu] = 10^{-4} M. Charges have been omitted.

Figure S9. Representation of pCu for the Cu^{2+}-L systems, as a function of pH, calculated from potentiometric titrations in 0.15 M NaCl, [L] = 10^{-5} M; [Cu^{2+}] = 10^{-6} M.

Figure S10. Detail of the chains formed by hydrogen bonding between the $[Cu(H\mathbf{L1})Br_2]^+$ units, water molecules and bromide units.

Figure S11. Representation of the IC_{50} values of ligands **L1**-**L4** and **L6** for the Cu^{2+}-L systems, using the data from Table 6.

Figure S12. ^1H NMR spectrum of **L1** in D_2O.

Figure S13. ^{13}C NMR spectrum of **L1** in D_2O.

Figure S14. ^1H NMR spectrum of **L2** in D_2O.

Figure S15. ^{13}C NMR spectrum of **L2** in D_2O.

Figure S16. ^1H NMR spectrum of **L3** in D_2O.

Figure S17. ^{13}C NMR spectrum of **L3** in D_2O.

Figure S18. ^1H NMR spectrum of **L4** in D_2O.

Figure S19. ^{13}C NMR spectrum of **L4** in D_2O.

Figure S20. ^1H NMR spectrum of **L5** in D_2O.

Figure S21. ^{13}C NMR spectrum of **L5** in D_2O.

Figure S22. ^1H NMR spectrum of **L6** in D_2O.

Figure S23. ^{13}C NMR spectrum of **L6** in D_2O.

Figure S24. Experimental (top) and calculated (bottom) HR-ESI-Mass Spectra for the [**L1**+H]$^+$ system, in H_2O/CH_3OH (50/50 vol/vol).

Figure S25. Experimental (top) and calculated (bottom) HR-ESI-Mass Spectra for the [**L2**+H]$^+$ system, in H_2O/CH_3OH (50/50 vol/vol).

Figure S26. Experimental (top) and calculated (bottom) HR-ESI-Mass Spectra for the [**L3**+H]$^+$ system, in H_2O/CH_3OH (50/50 vol/vol).

Figure S27. Experimental (top) and calculated (bottom) HR-ESI-Mass Spectra for the [**L4**+H]$^+$ system, in H_2O/CH_3OH (50/50 vol/vol).

Figure S28: Experimental (top) and calculated (bottom) HR-ESI-Mass Spectra for the [**L5**+D]$^+$ system, in H_2O/CH_3OH (50/50 vol/vol).

Figure S29. Experimental (top) and calculated (bottom) HR-ESI-Mass Spectra for the [**L6**+D]$^+$ system, in H_2O/CH_3OH (50/50 vol/vol).

II. Tables

Table S1. Crystallographic data for crystals (**1**) and (**2**).

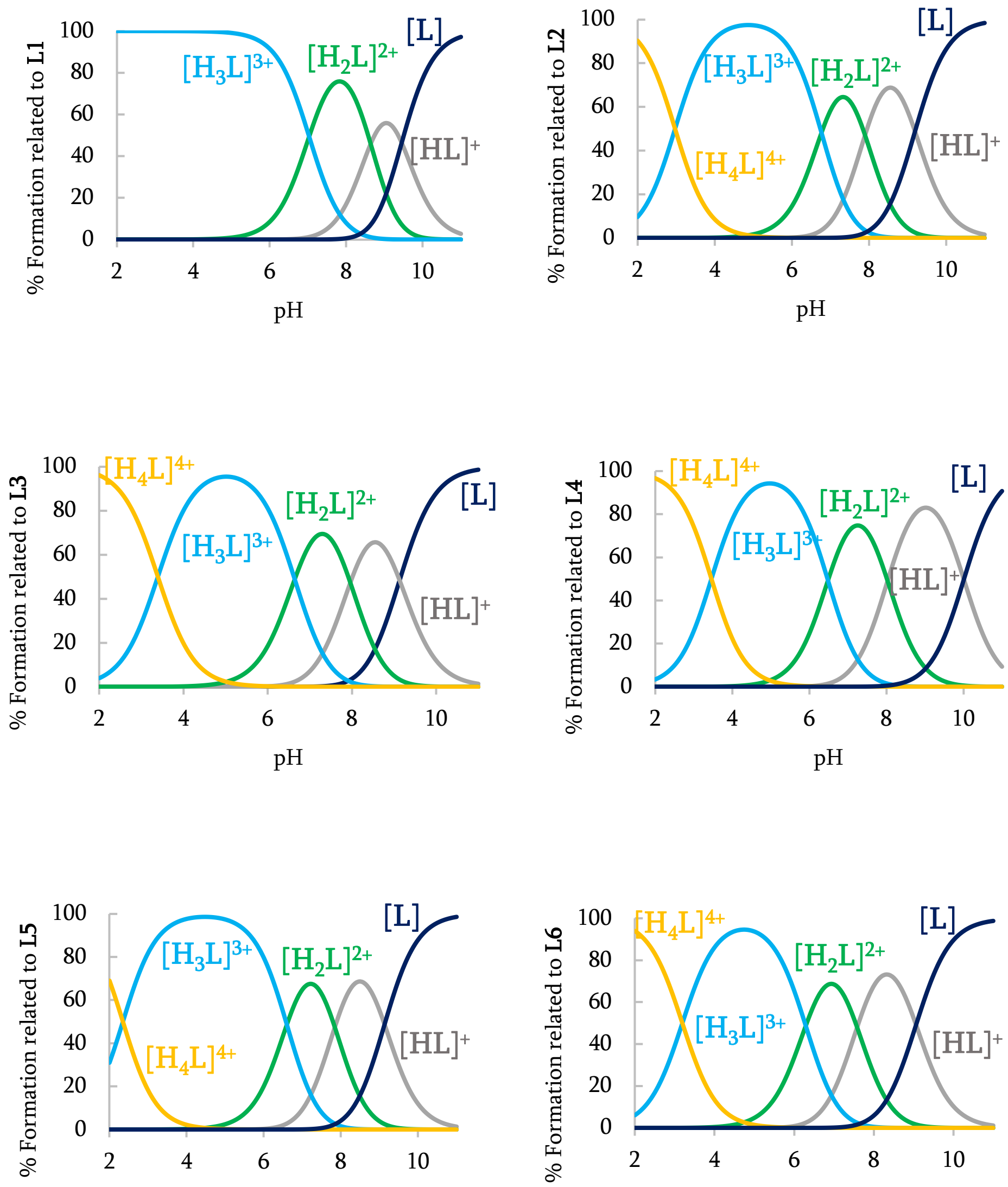

Figure S1. Distribution diagrams for **L1**-**L6** in the pH range 2.5-11.0.

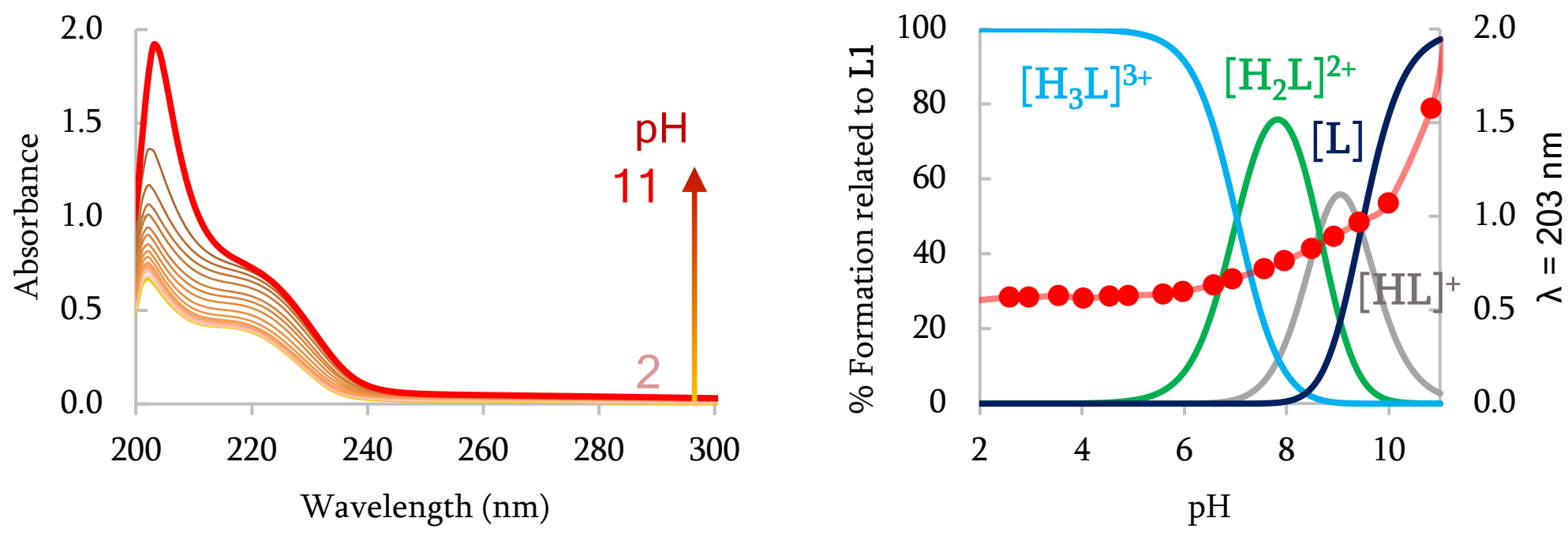

Figure S2. Variation with the pH of the UV spectra of **L1** (left) and variation of the absorbance at 203 nm overlapped with the distribution diagram (right).

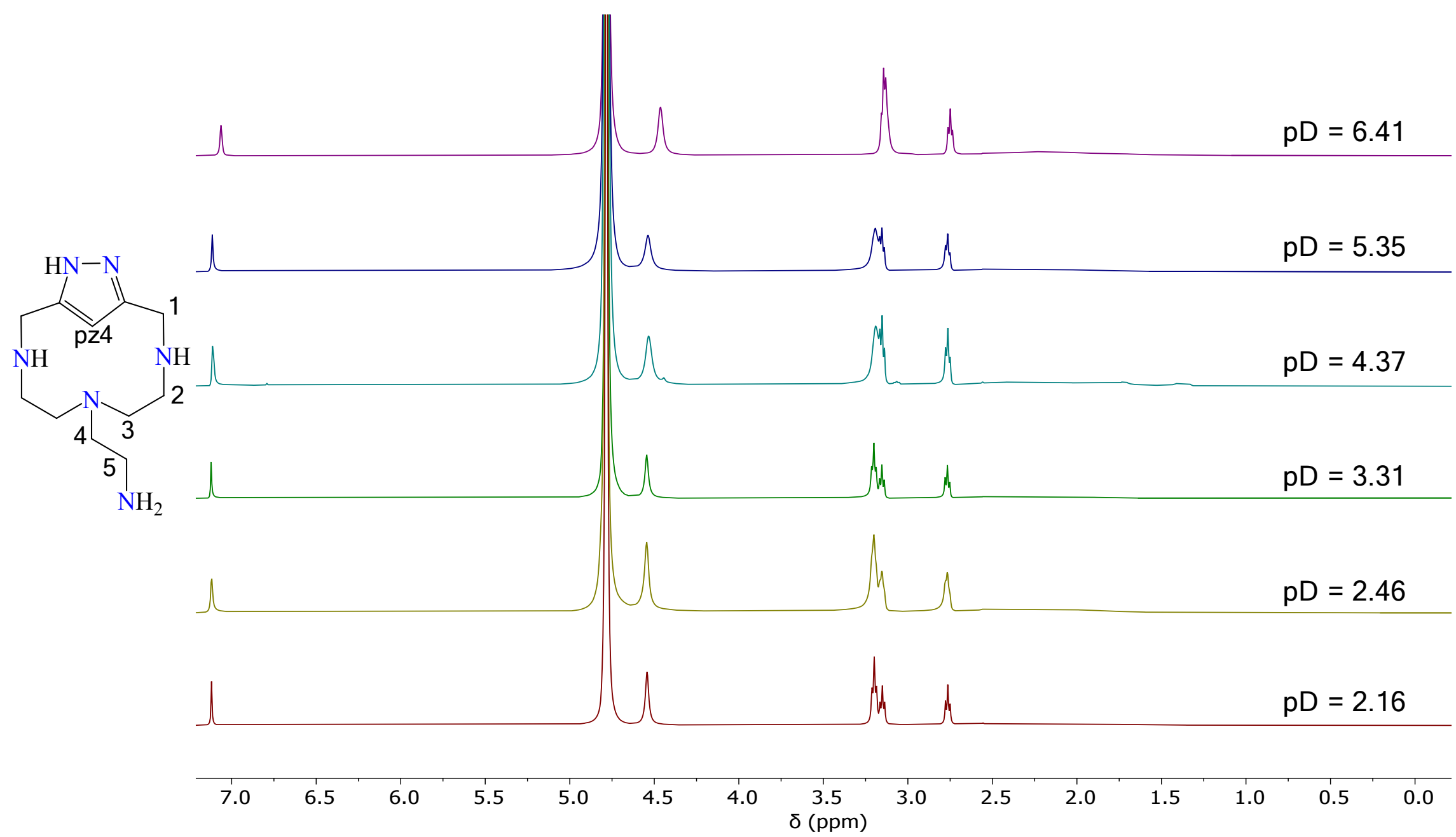

Figure S3. Variation of the chemical shifts of **L1** in the pD 2.16-6.41.

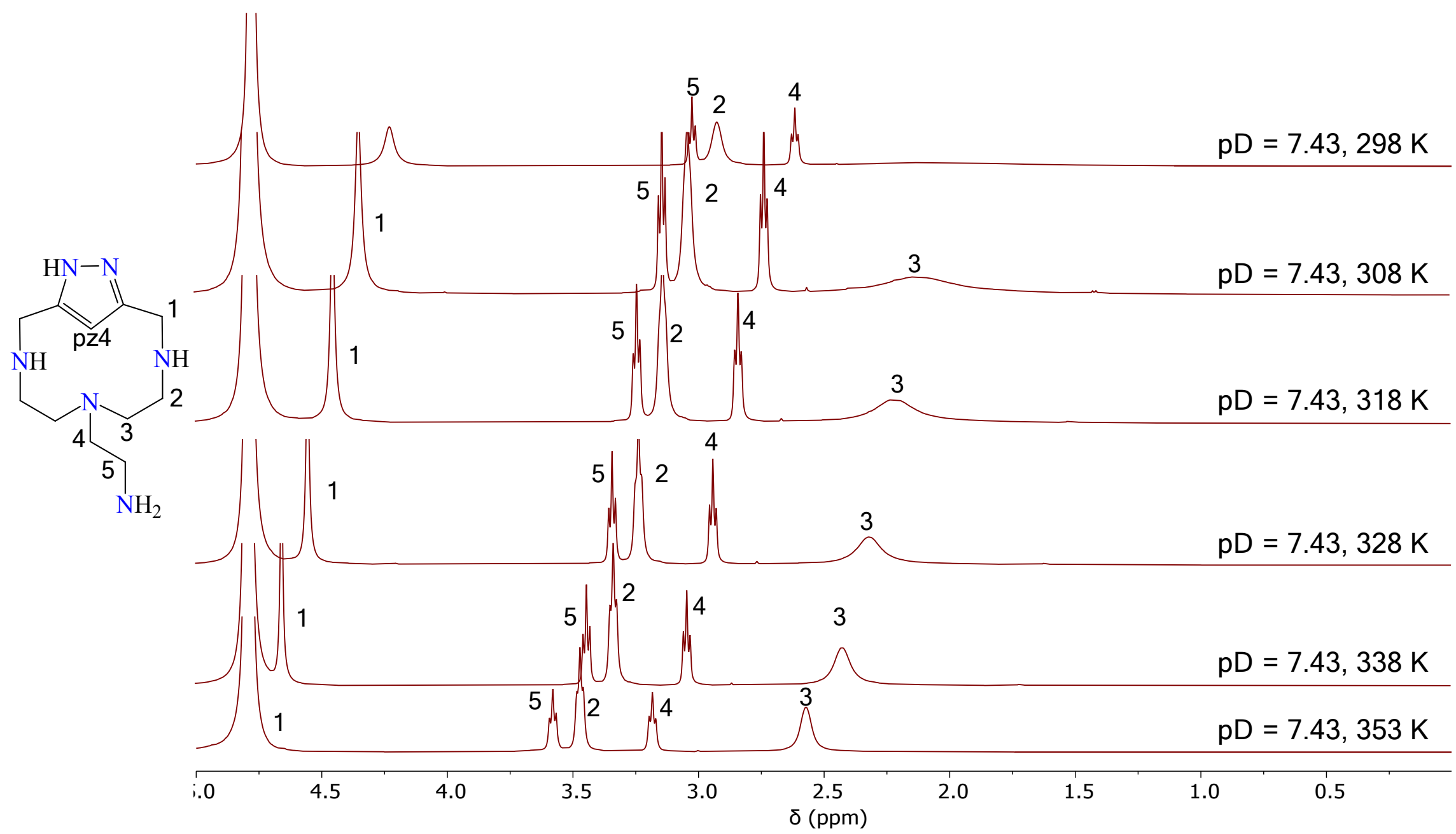

Figure S4. ^{1}H NMR spectra of **L1** at 298 K (top) and 353 K (bottom).

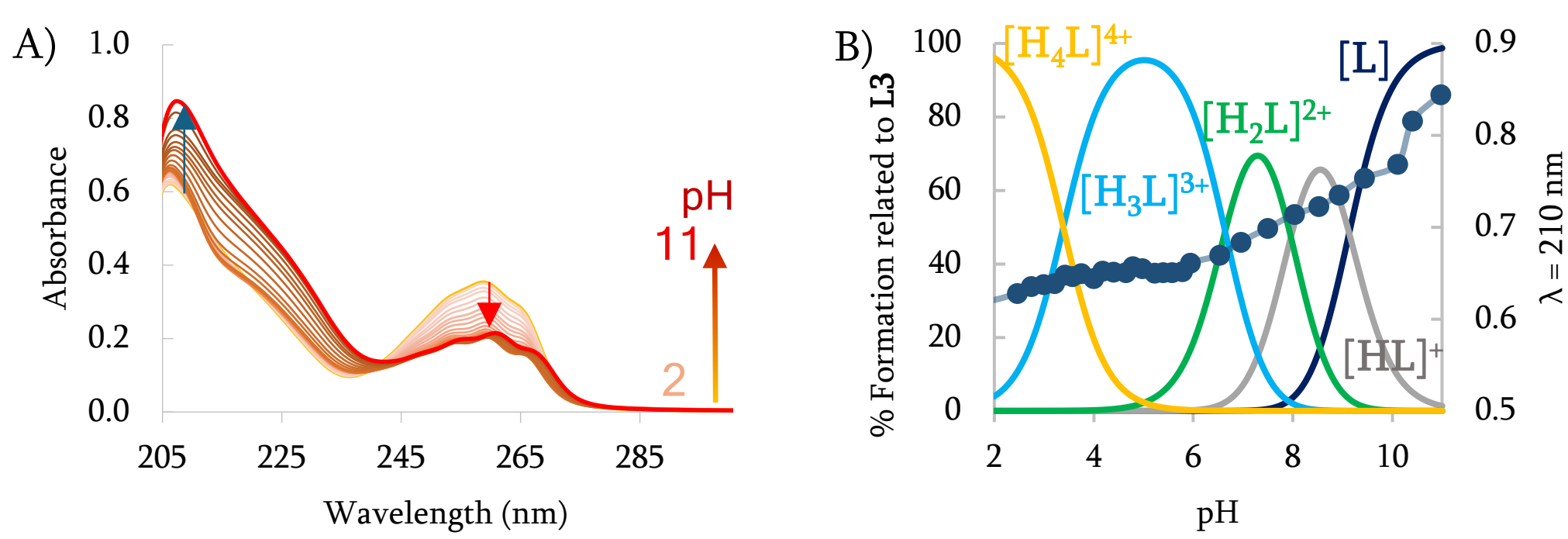

Figure S5. A) Variation of the UV-Vis spectra of **L3** with pH. B) Variation of the band centered at 210 nm with pH overlapped with the distribution diagram. [L] = 1 × 10^{-4} M.

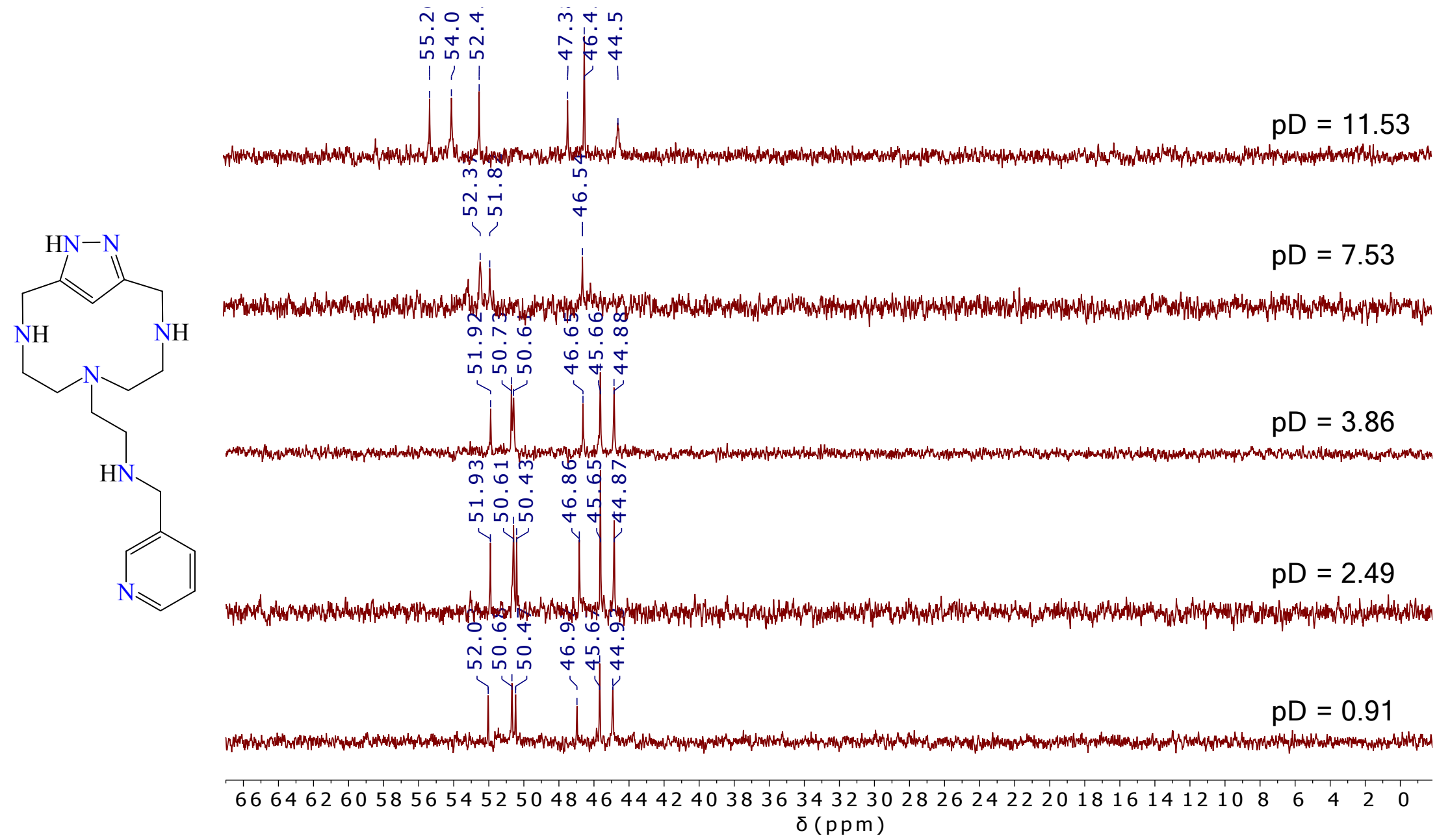

Figure S6. Variation of the ^{13}C NMR spectra of **L3** with pH.

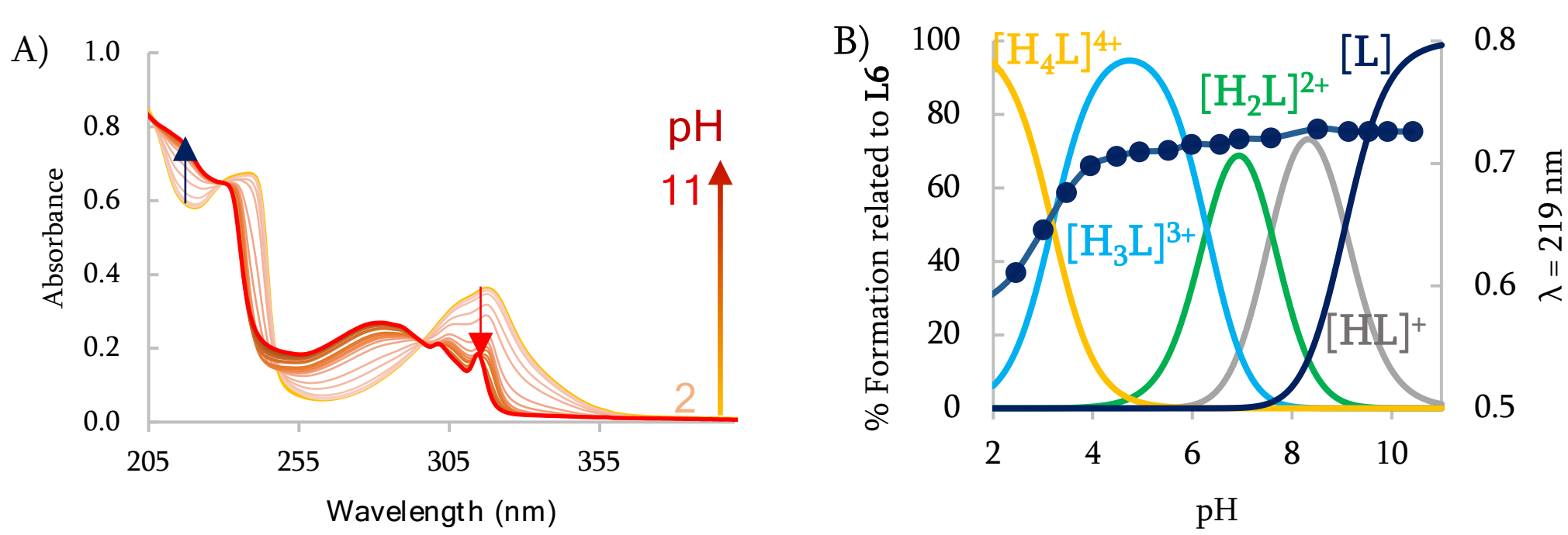

Figure S7. A) Variation of the UV-Vis spectra of **L6** with pH. B) Variation of the band centered at 219 nm with pH overlapped with the distribution diagram. [L] = 1 × 10^{-4} M.

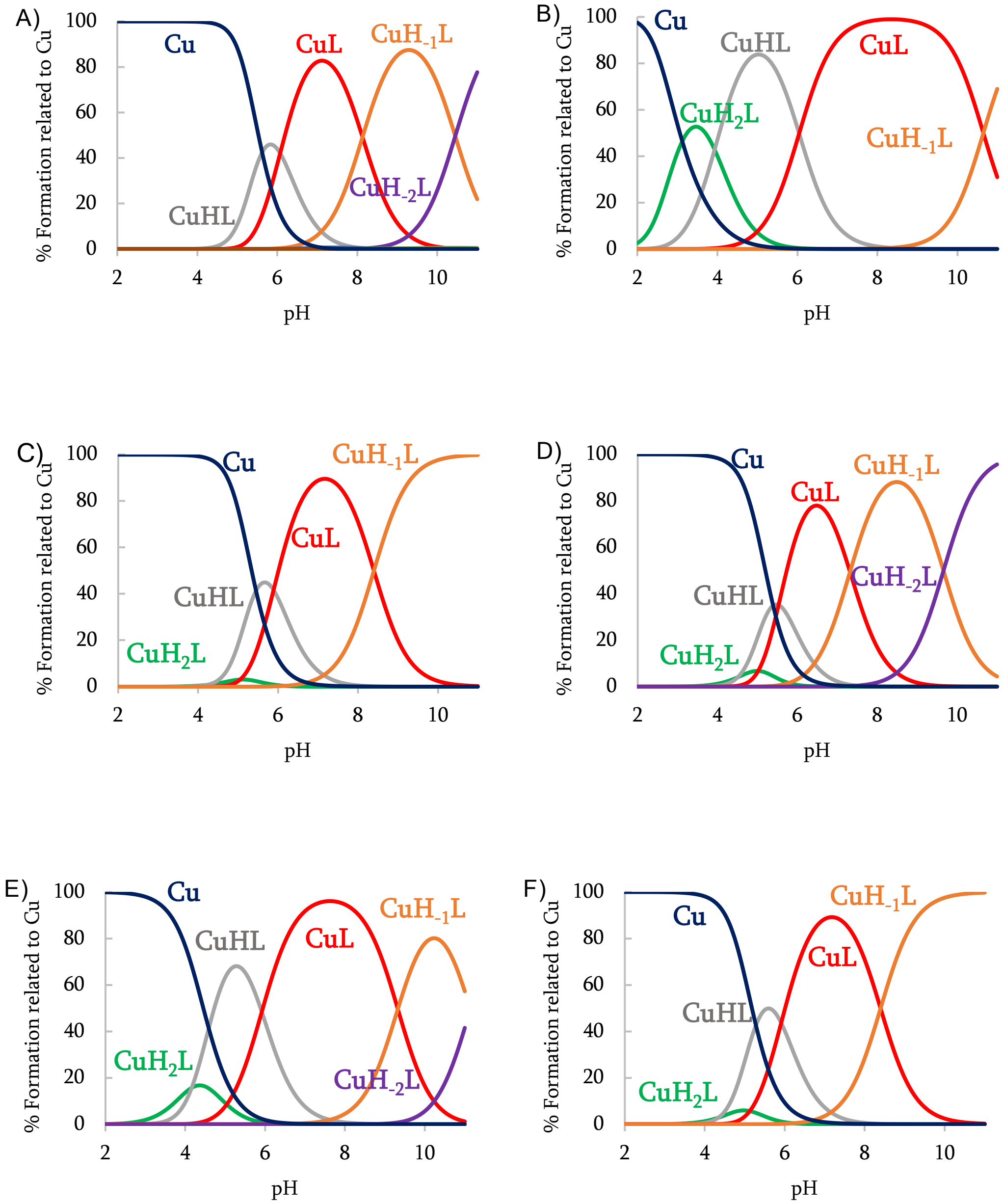

Figure S8. Distribution diagrams of the systems A) Cu(II)-**L1**, B) Cu(II)-**L2**, C) Cu(II)-**L3**, D) Cu(II)-**L4,** E) Cu(II)-**L5** and F) Cu(II)-**L6** related to Cu(II) in 1:1 molar ratio calculated from the potentiometric constants. In NaCl 0.15 M. [L] = [Cu] = 10^{-4} M. Charges have been omitted.

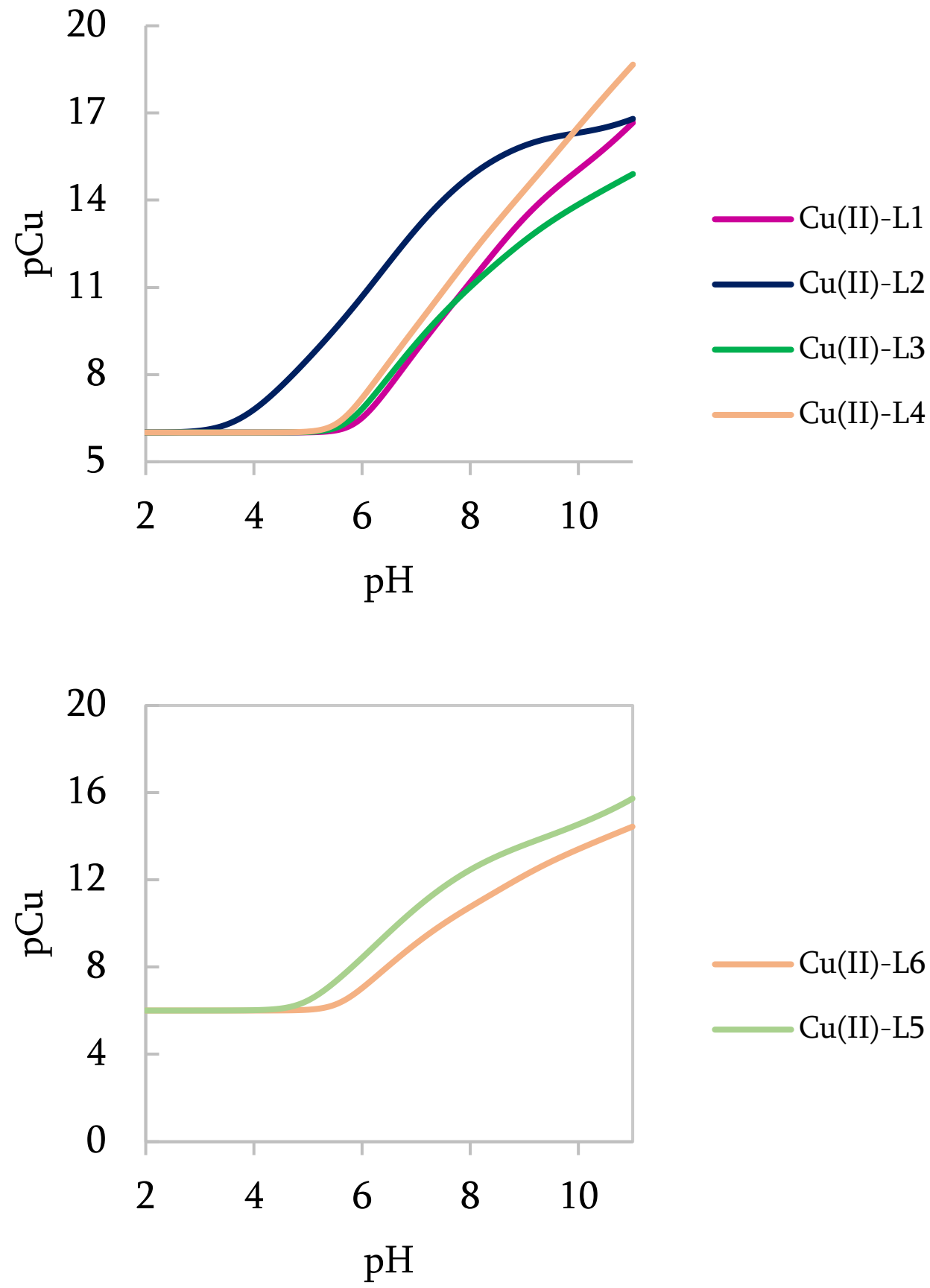

Figure S9. Representation of pCu for the Cu^{2+}-L systems, as a function of pH, calculated from potentiometric titrations in 0.15 M NaCl, [L] = 10^{-5} M; [Cu^{2+}] = 10^{-6} M.

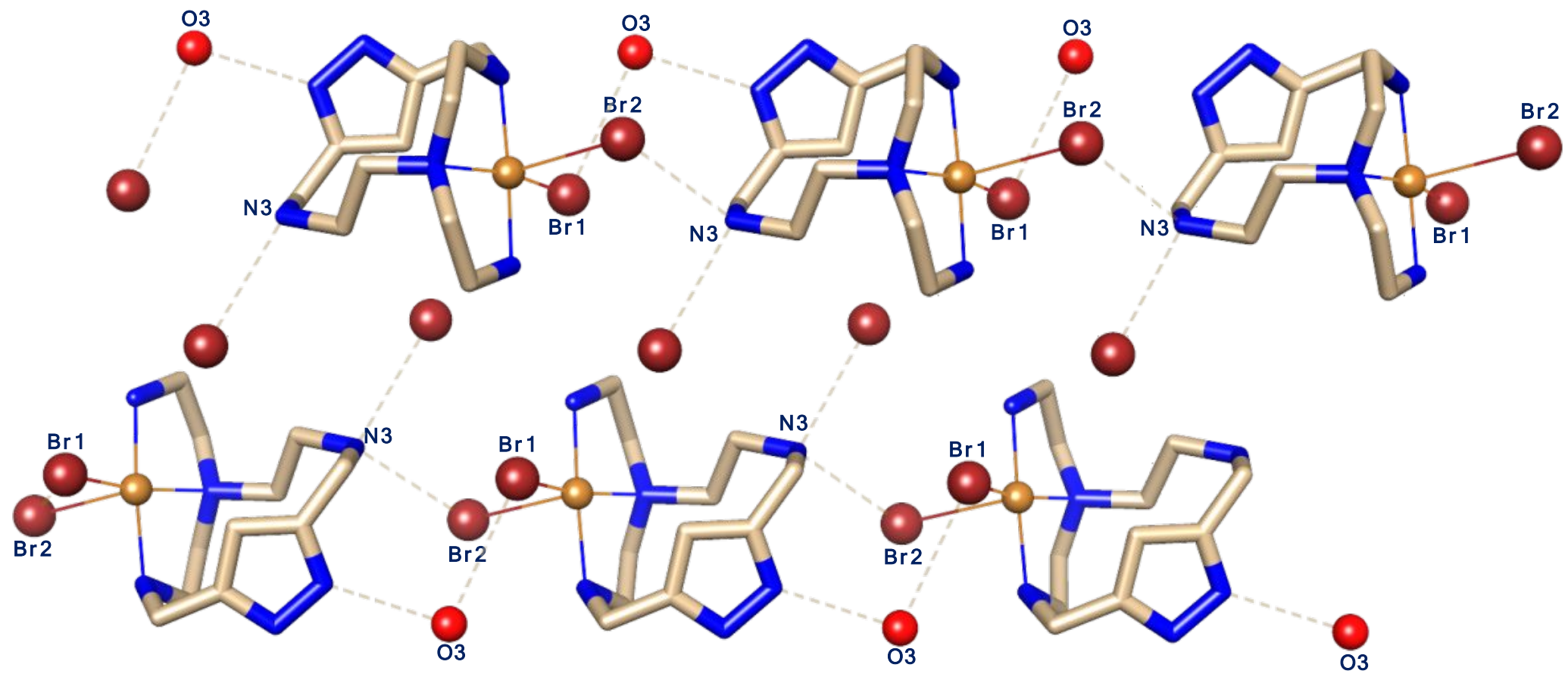

Figure S10. Detail of the chains formed by hydrogen bonding between the $[Cu(H\mathbf{L1})Br_2]^+$ units, water molecules and bromide units.

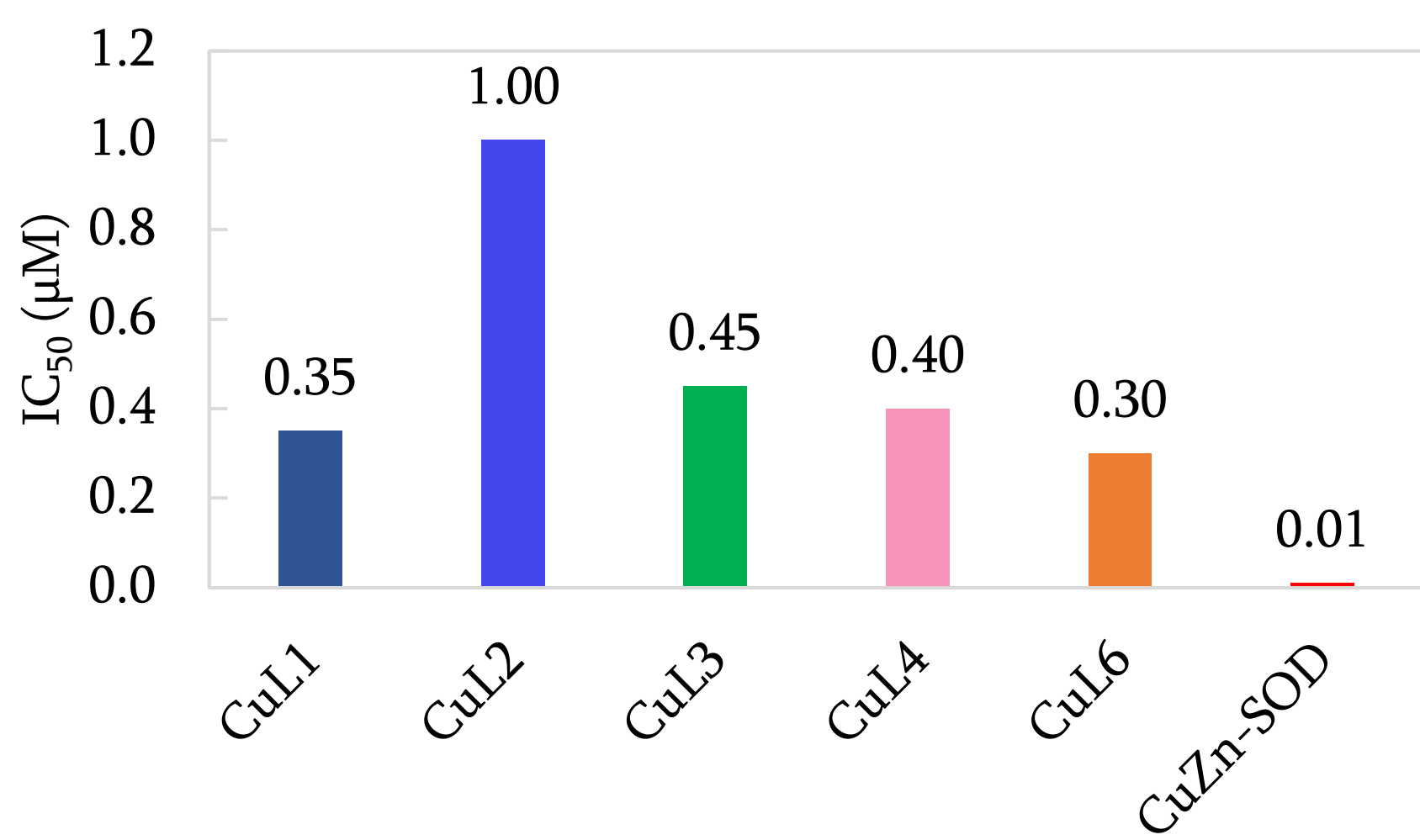

Figure S11. Representation of the IC_{50} values of ligands **L1-L4** and **L6** for the Cu^{2+}-L systems, using the data from Table 6.

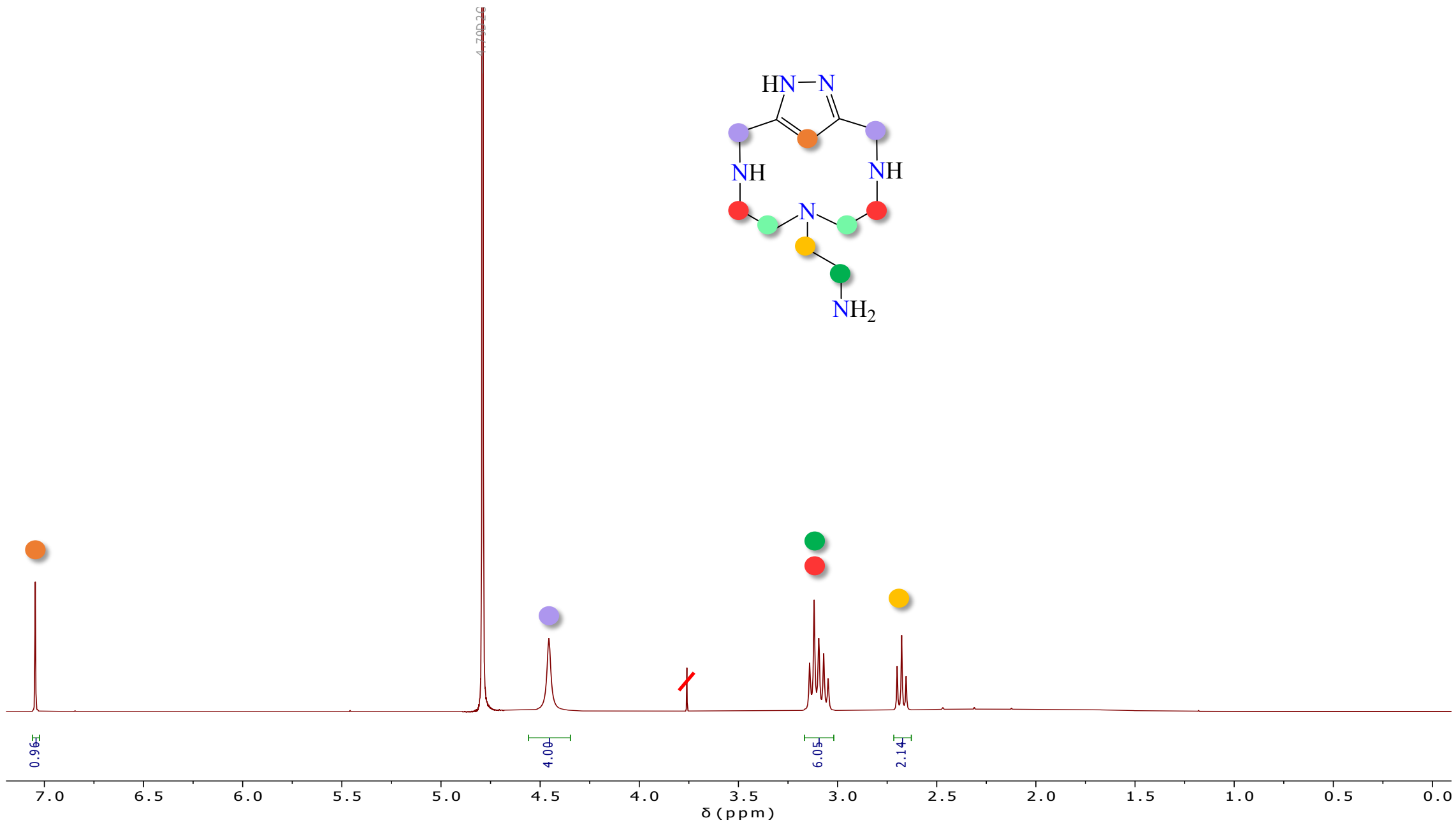

Figure S12. ^{1}H NMR spectrum of **L1** in D_2O.

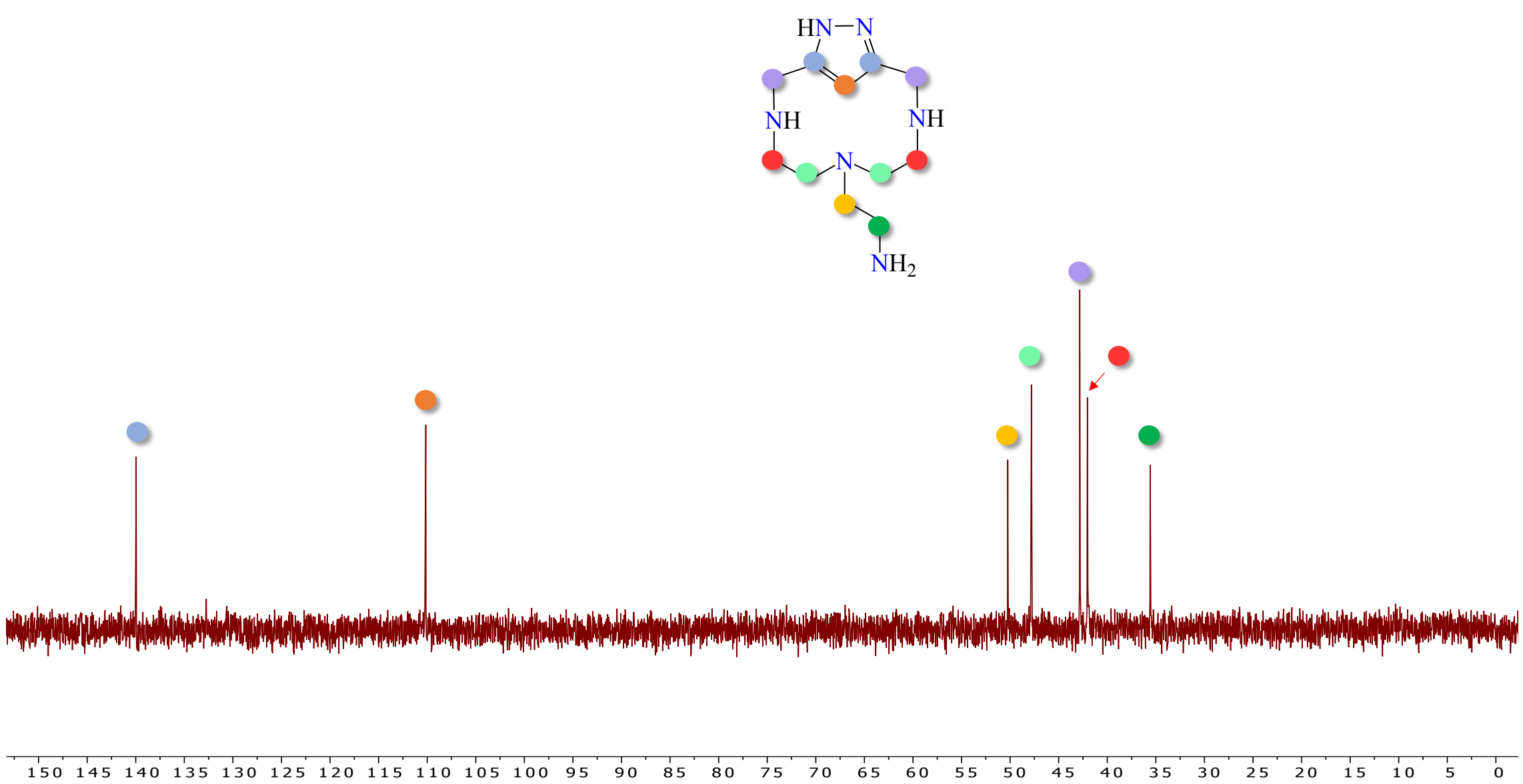

Figure S13. ^{13}C NMR spectrum of **L1** in D_2O.

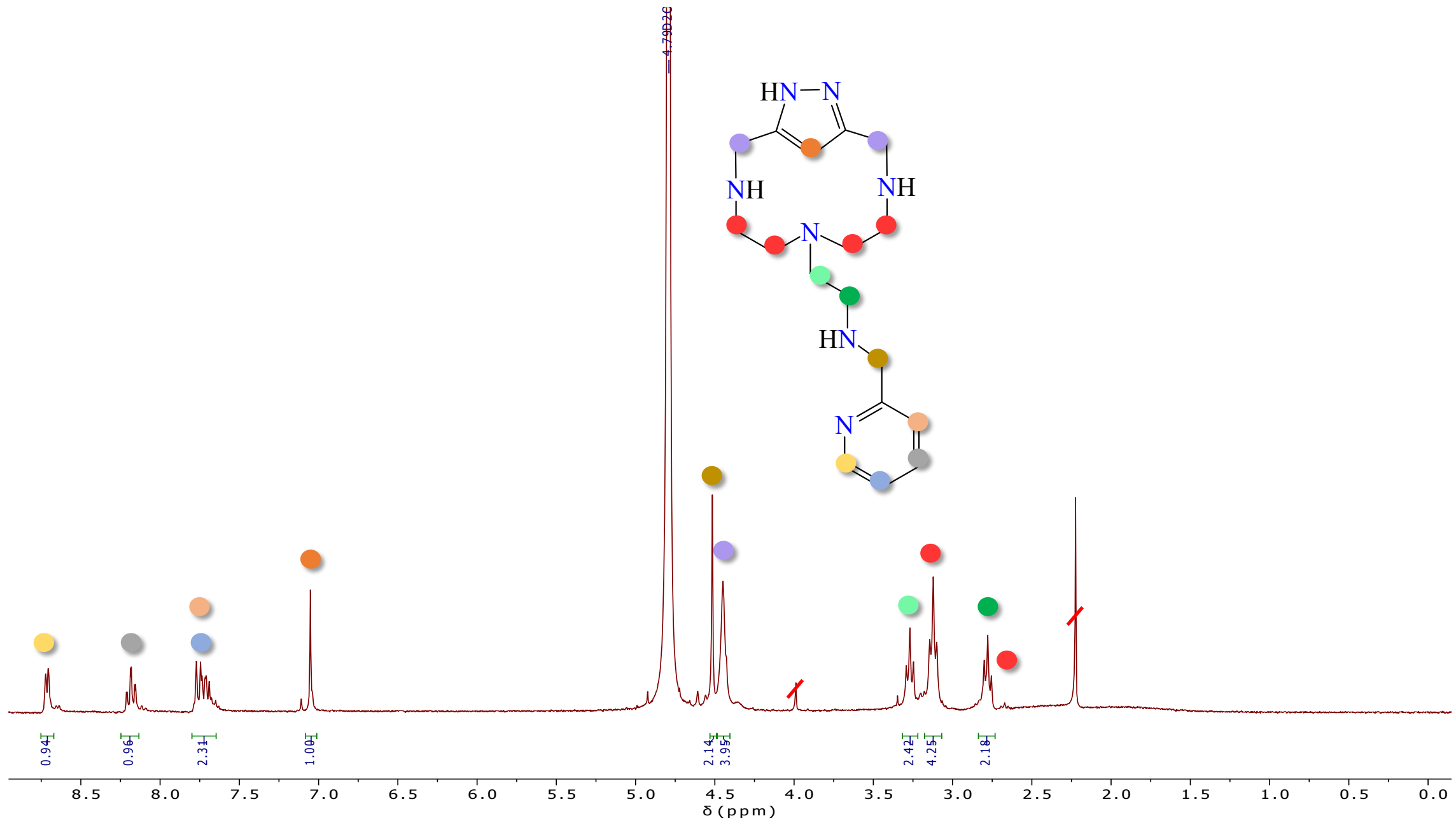

Figure S14. ^{1}H NMR spectrum of **L2** in D_2O.

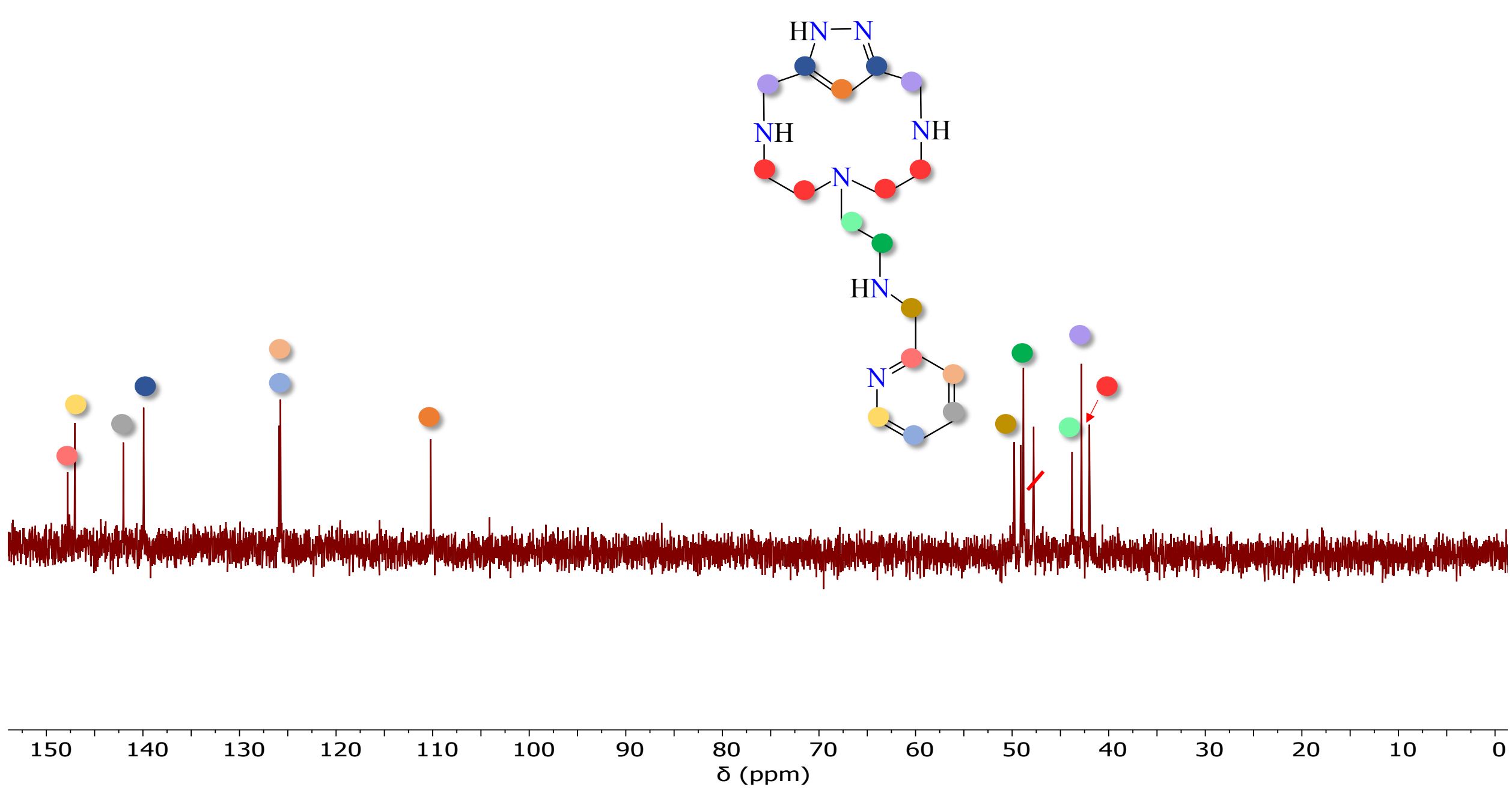

Figure S15. ^{13}C NMR spectrum of **L2** in D_2O.

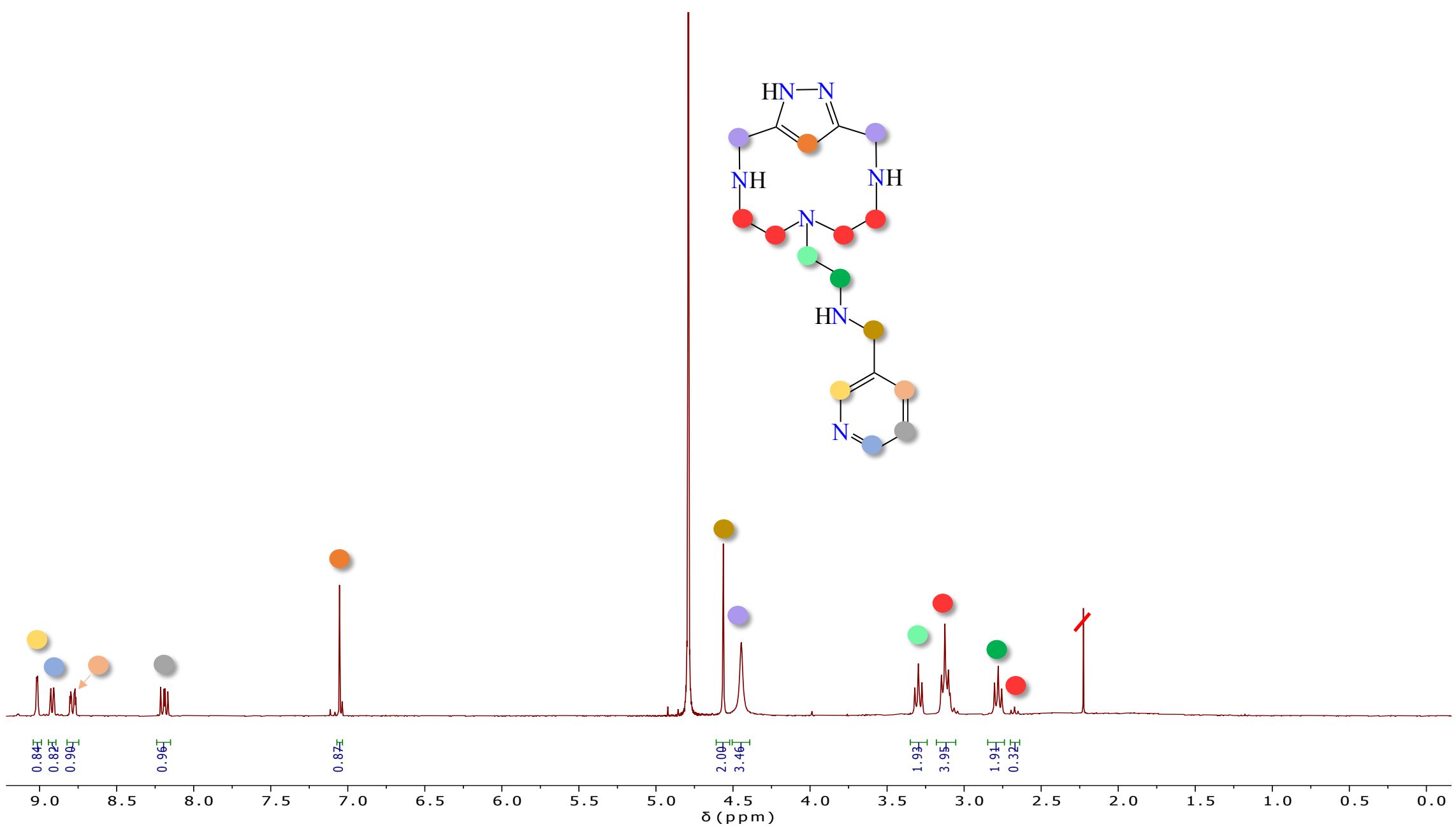

Figure S16. ^{1}H NMR spectrum of **L3** in D_2O.

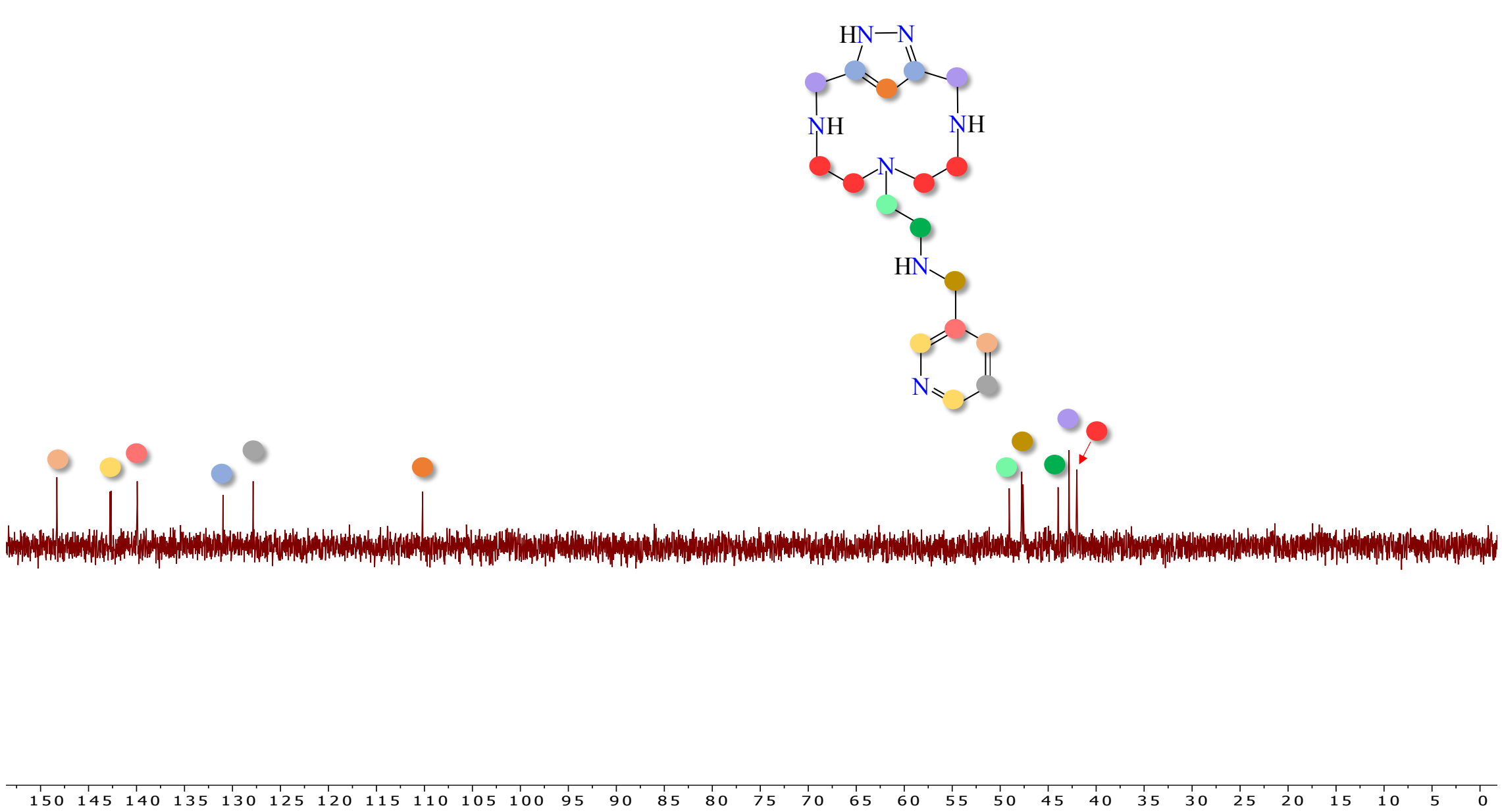

Figure S17. ^{13}C NMR spectrum of **L3** in D_2O.

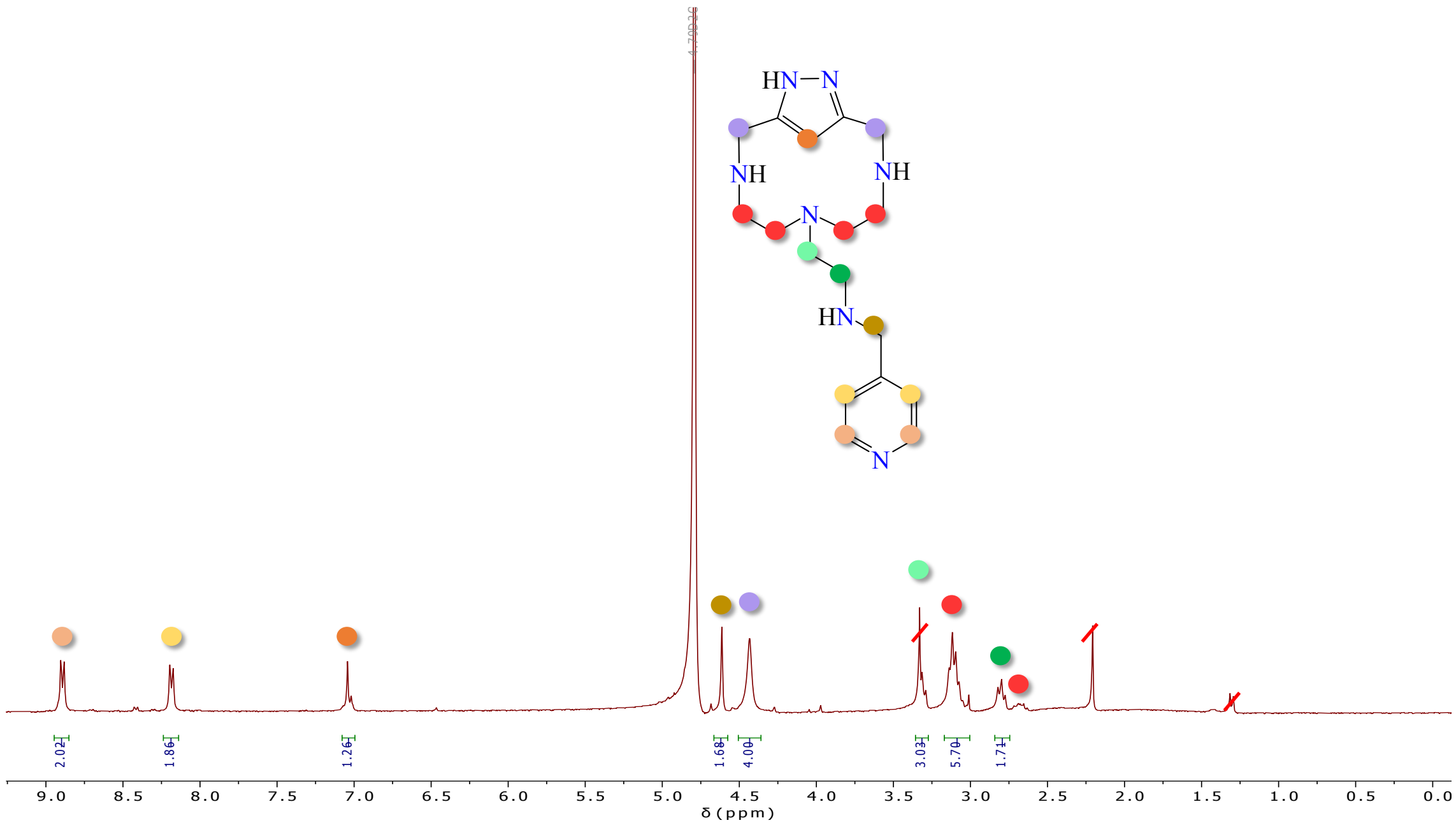

Figure S18. ^{1}H NMR spectrum of **L4** in D_2O.

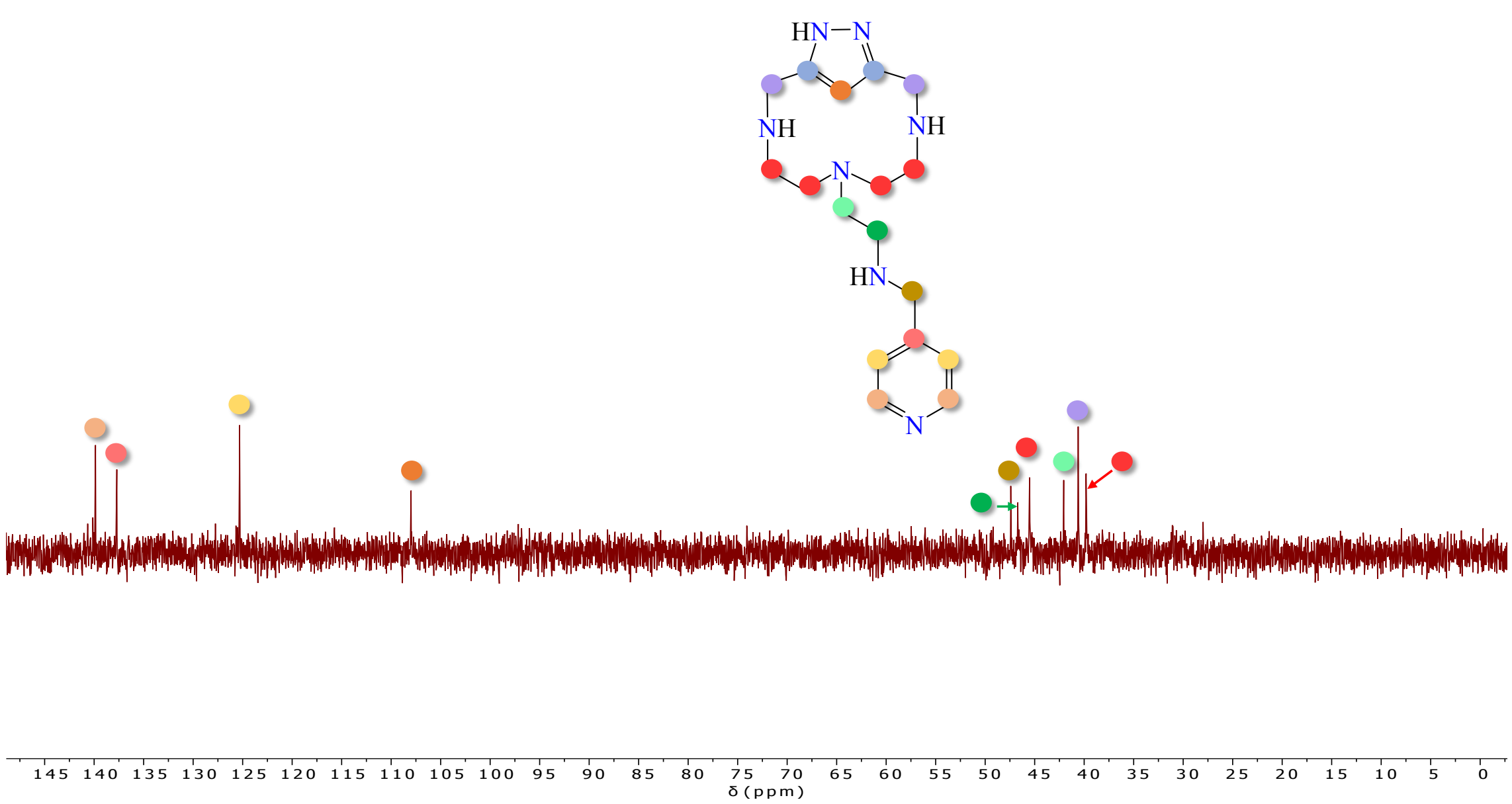

Figure S19. ^{13}C NMR spectrum of **L4** in D_2O.

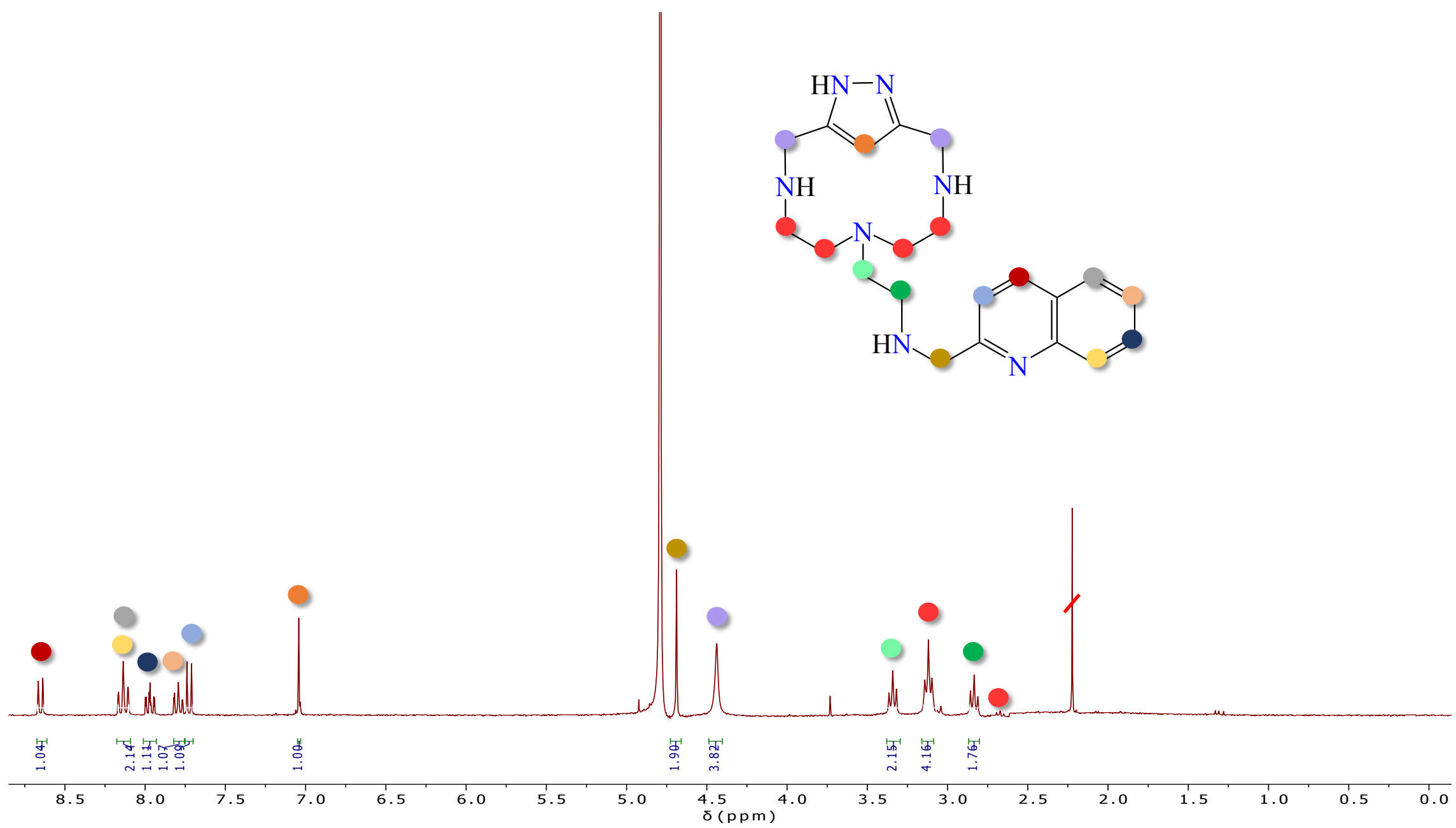

Figure S20. ^{1}H NMR spectrum of **L5** in D_2O.

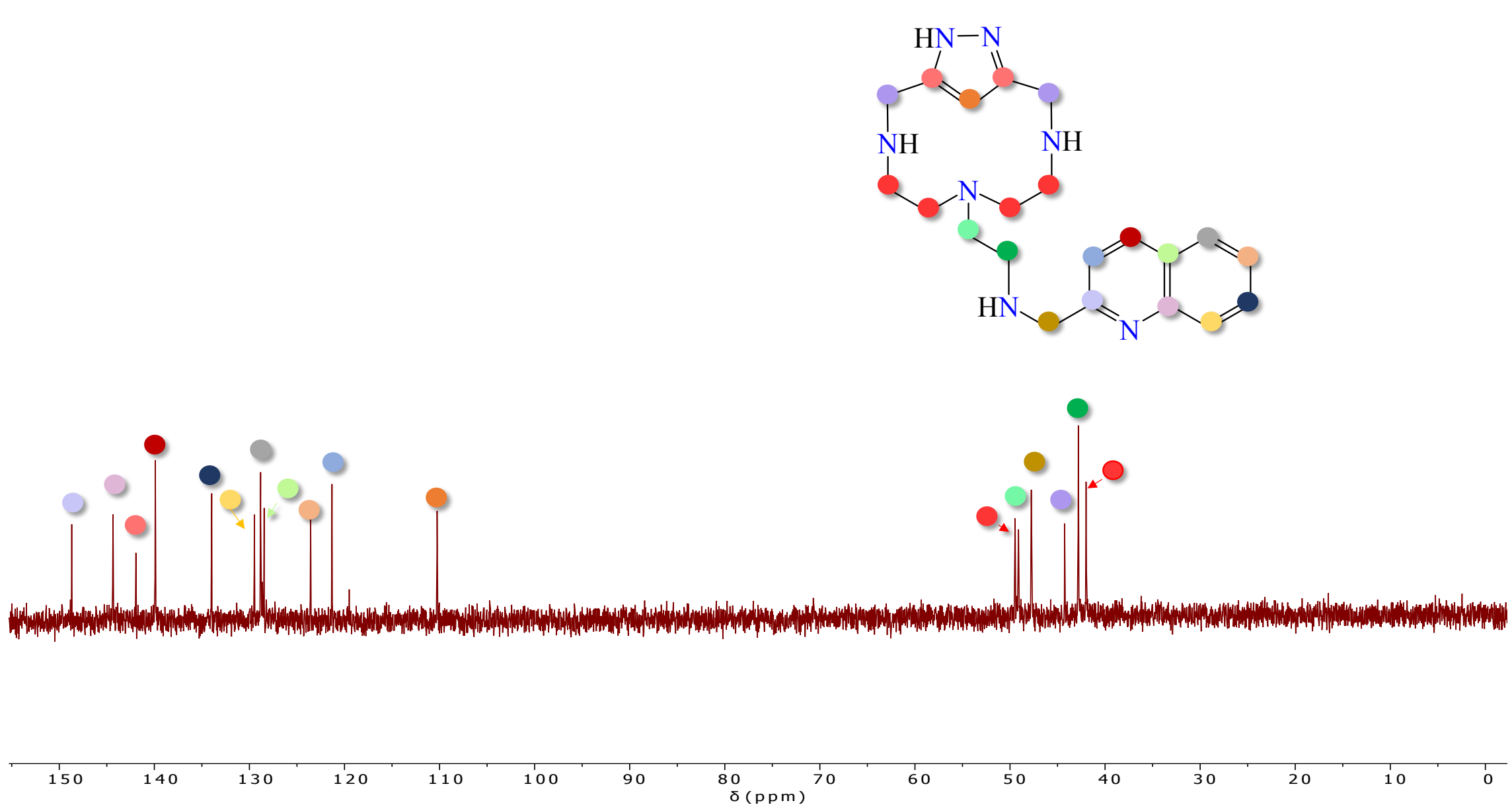

Figure S21. ^{13}C NMR spectrum of **L5** in D_2O.

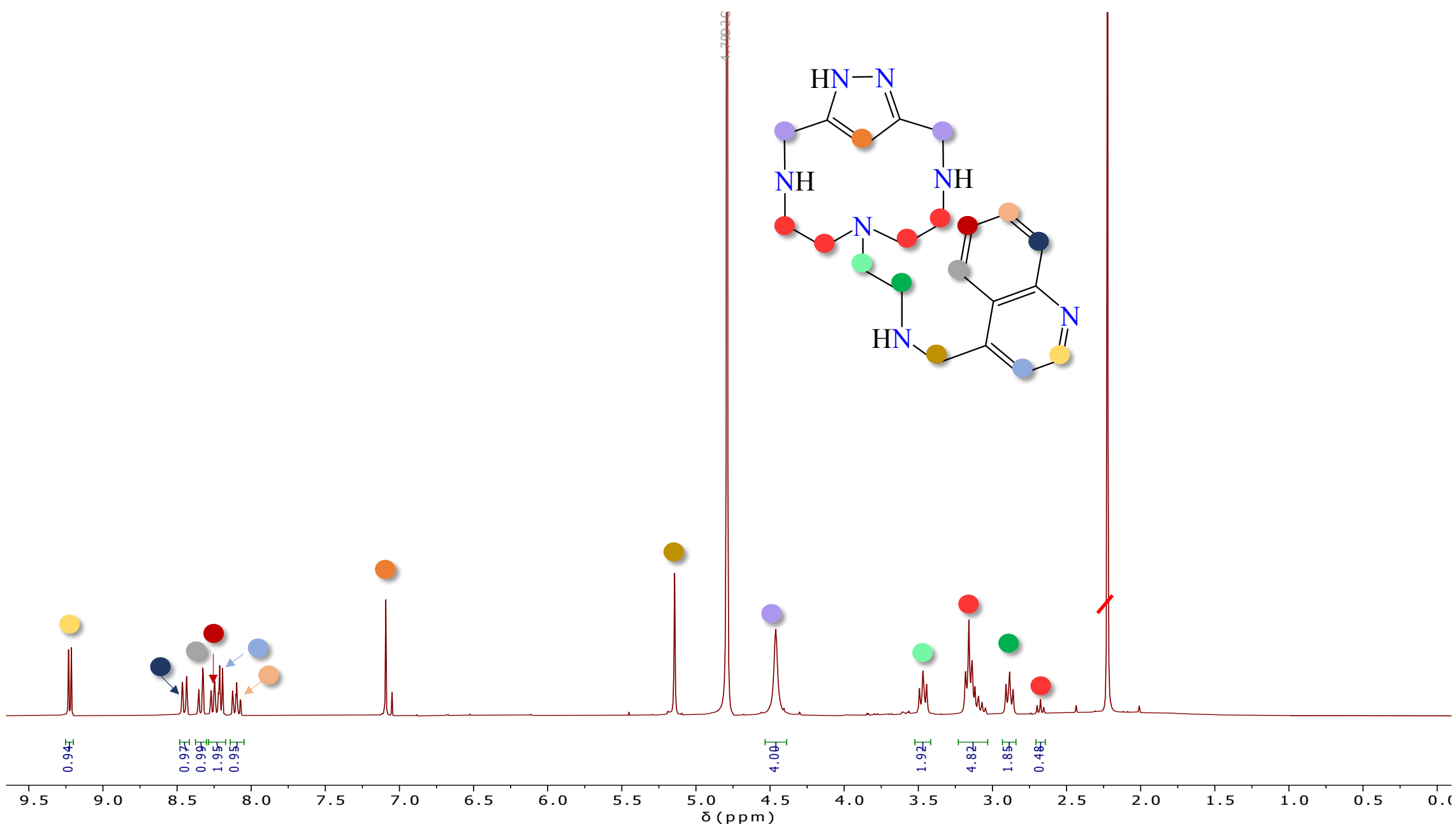

Figure S22. ^{1}H NMR spectrum of **L6** in D_2O.

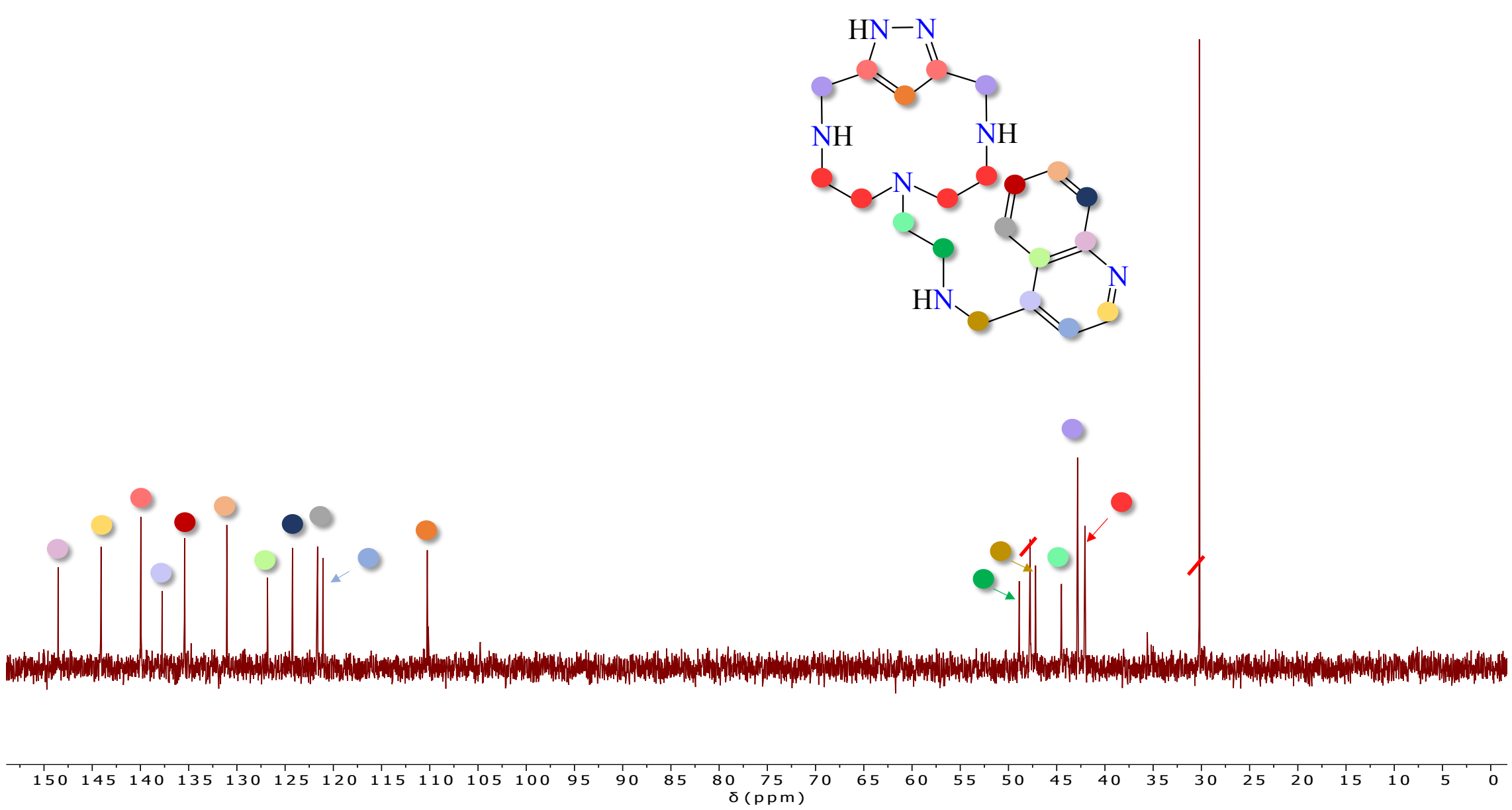

Figure S23. ^{13}C NMR spectrum of **L6** in D_2O.

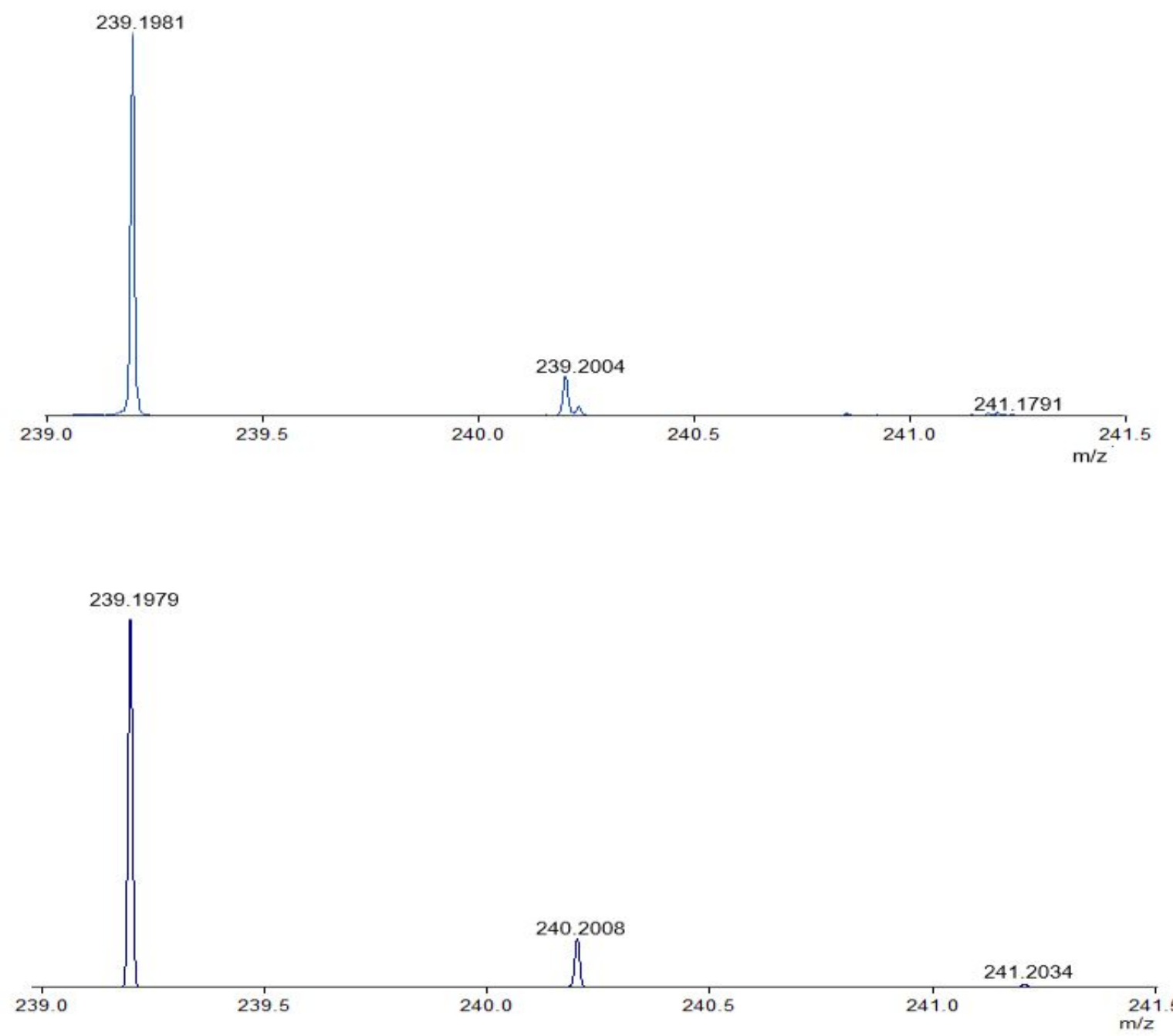

Figure S24. Experimental (top) and calculated (bottom) HR-ESI-Mass Spectra for the [**L1**+H]$^+$ system, in H_2O/CH_3OH (50/50 vol/vol).

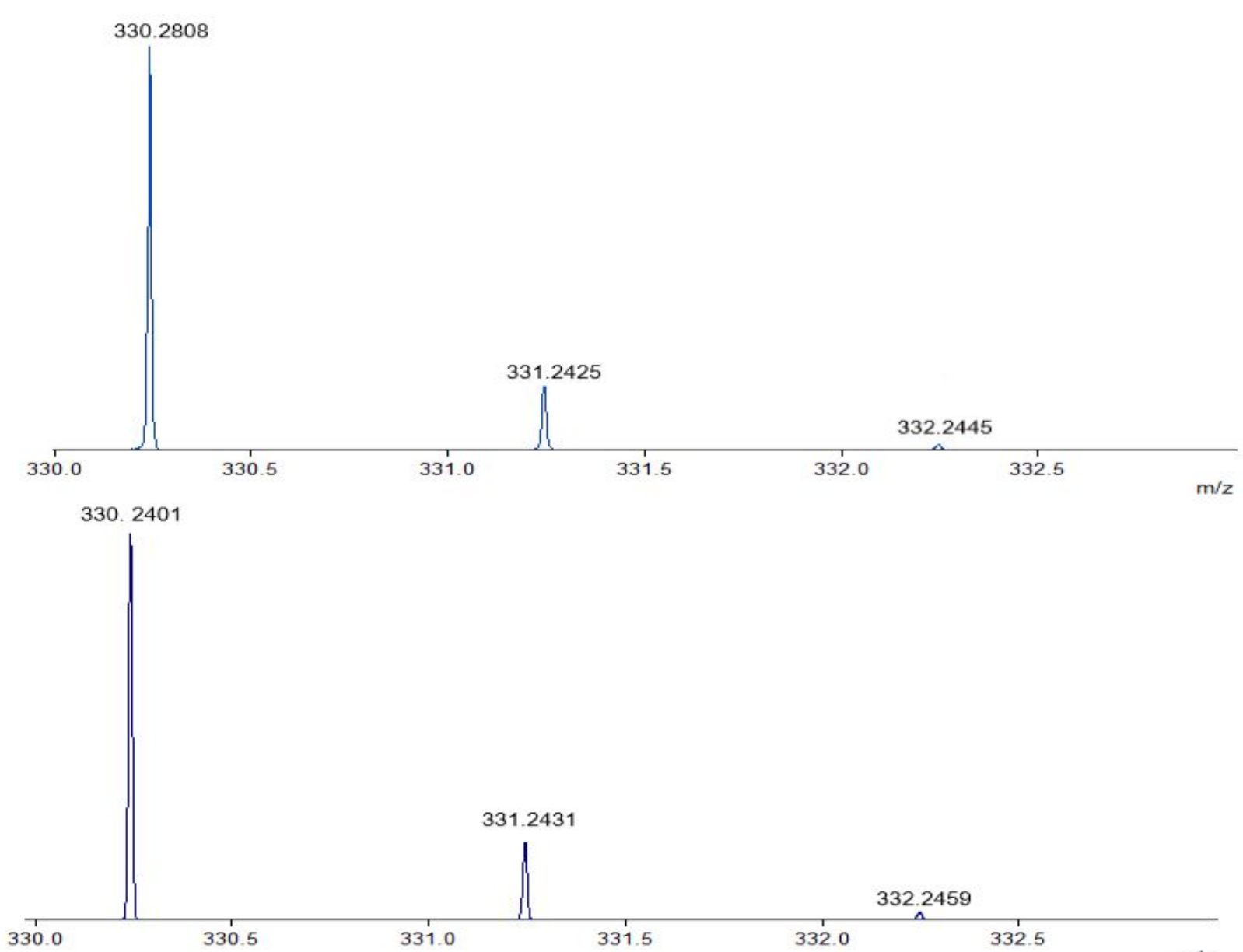

Figure S25. Experimental (top) and calculated (bottom) HR-ESI-Mass Spectra for the [**L2**+H]$^+$ system, in H_2O/CH_3OH (50/50 vol/vol).

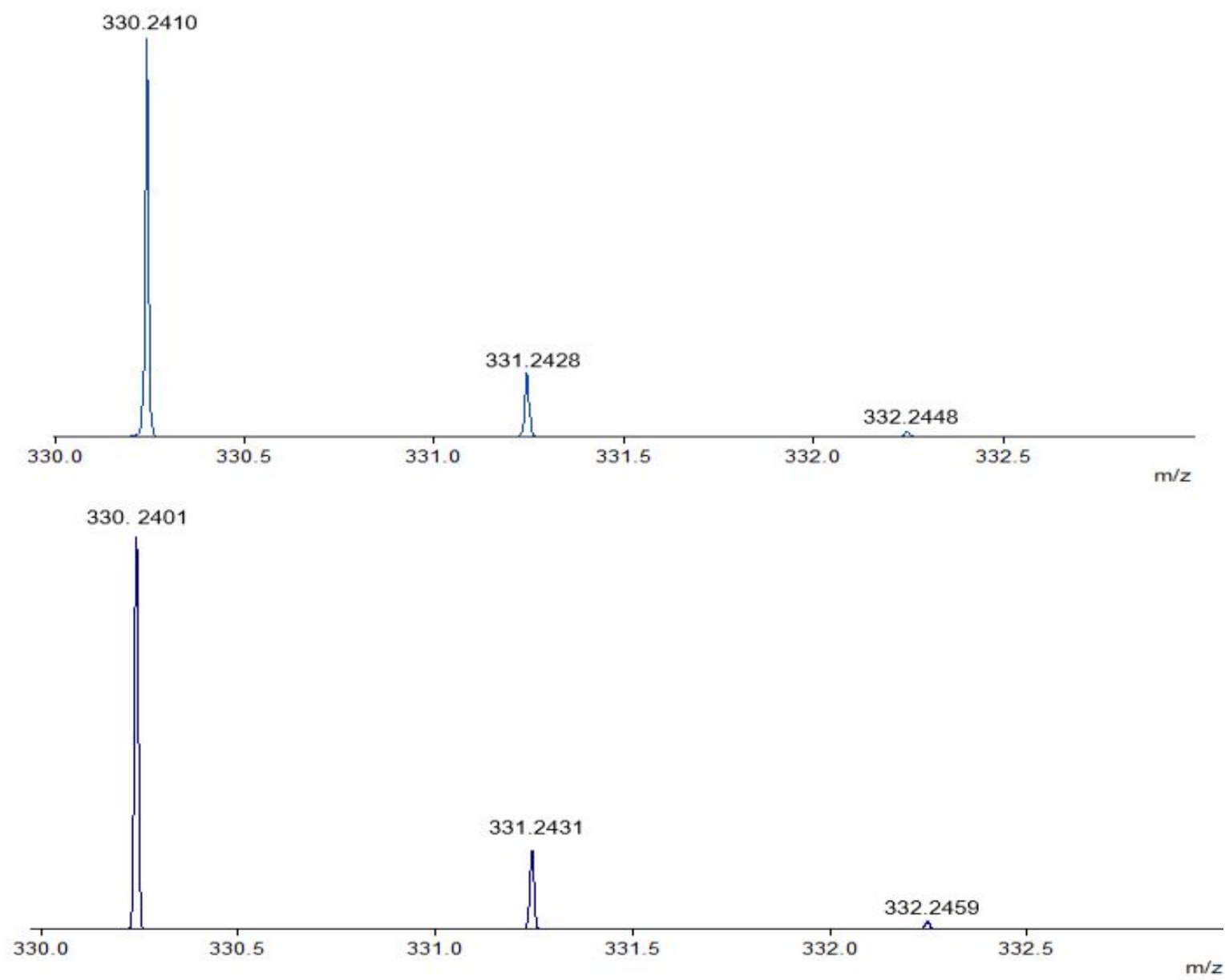

Figure S26. Experimental (top) and calculated (bottom) HR-ESI-Mass Spectra for the [**L3**+H]$^+$ system, in H_2O/CH_3OH (50/50 vol/vol).

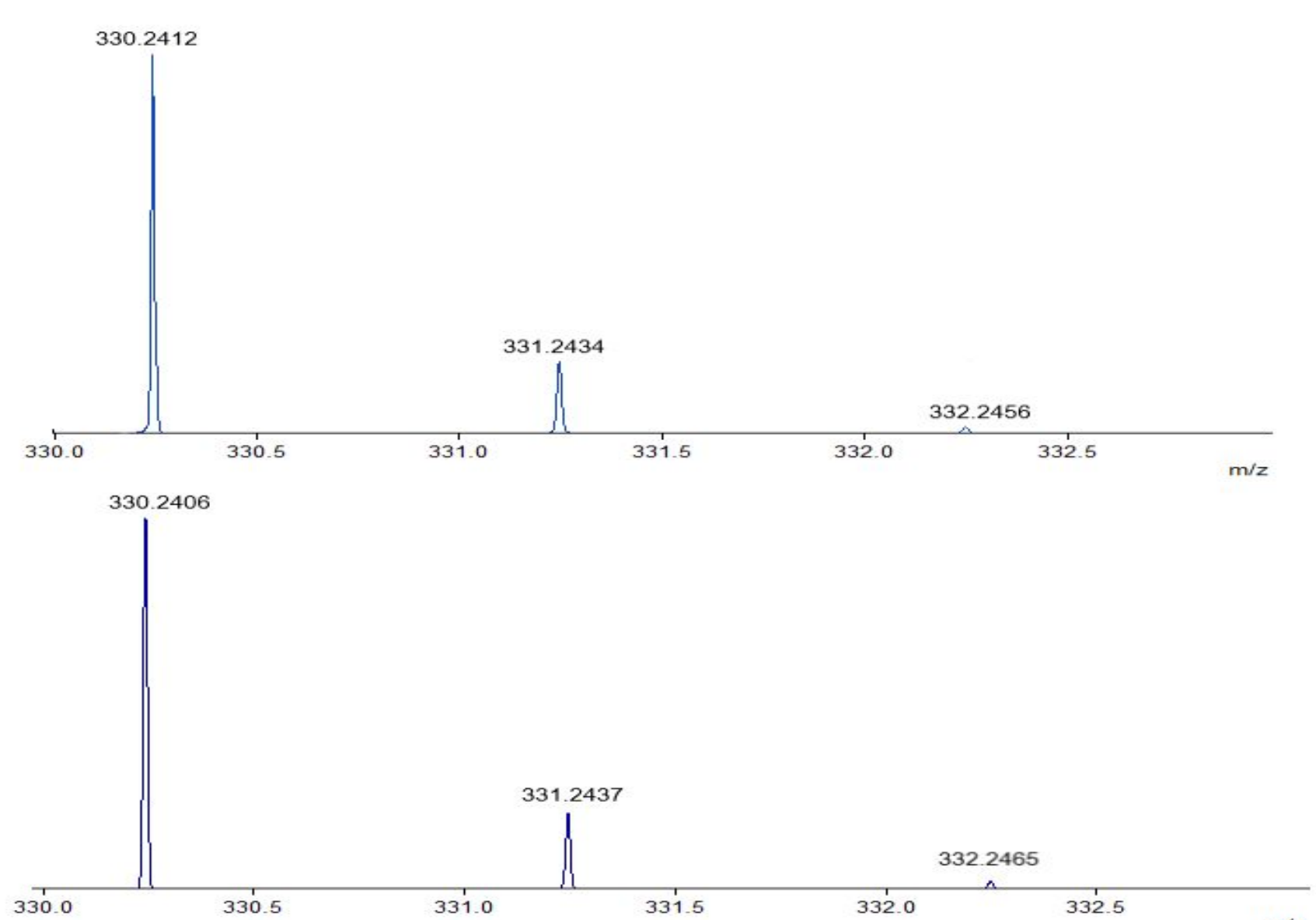

Figure S27. Experimental (top) and calculated (bottom) HR-ESI-Mass Spectra for the [**L4**+H]$^+$ system, in H_2O/CH_3OH (50/50 vol/vol).

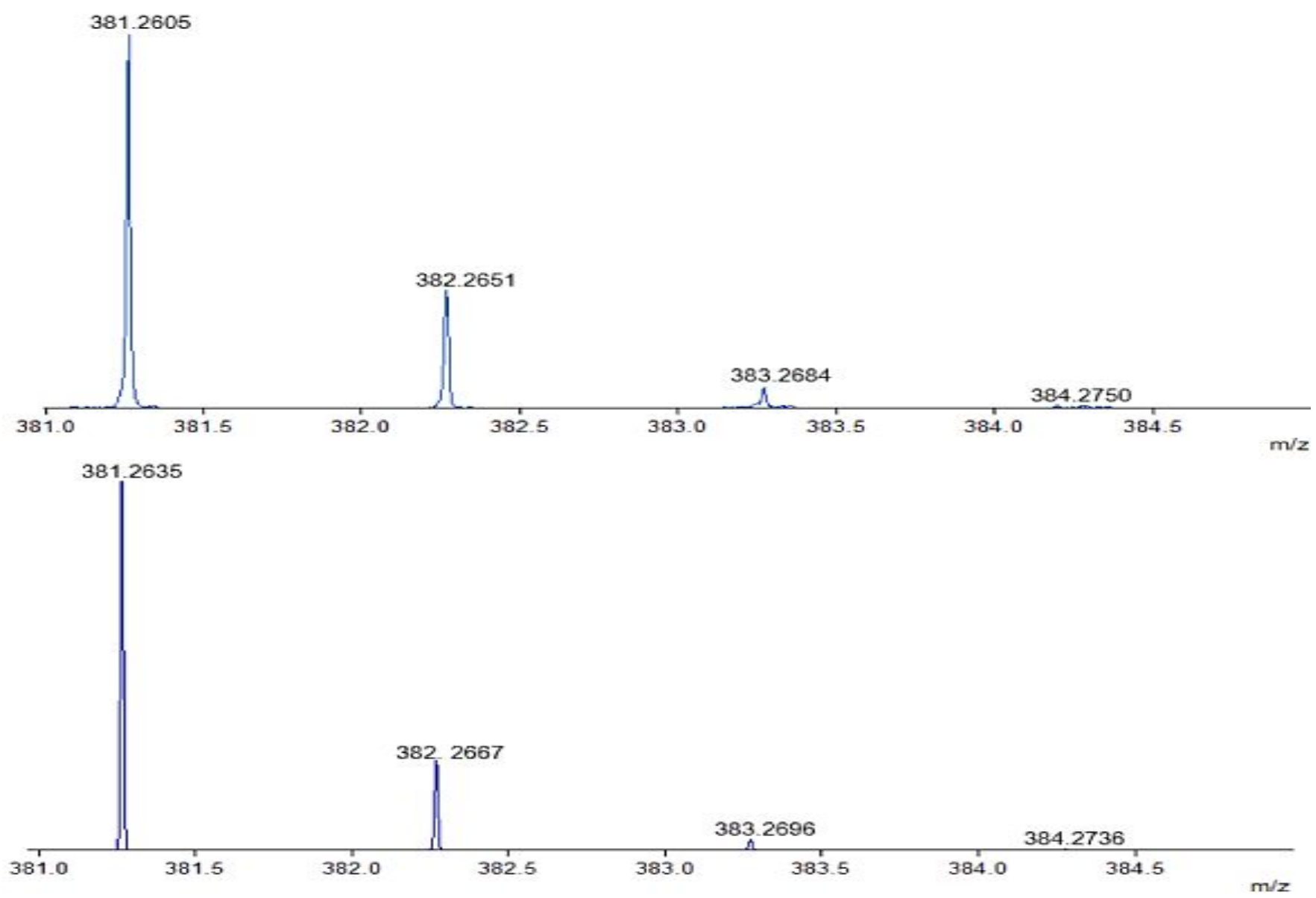

Figure S28. Experimental (top) and calculated (bottom) HR-ESI-Mass Spectra for the [**L5**+D]$^+$ system, in H_2O/CH_3OH (50/50 vol/vol).

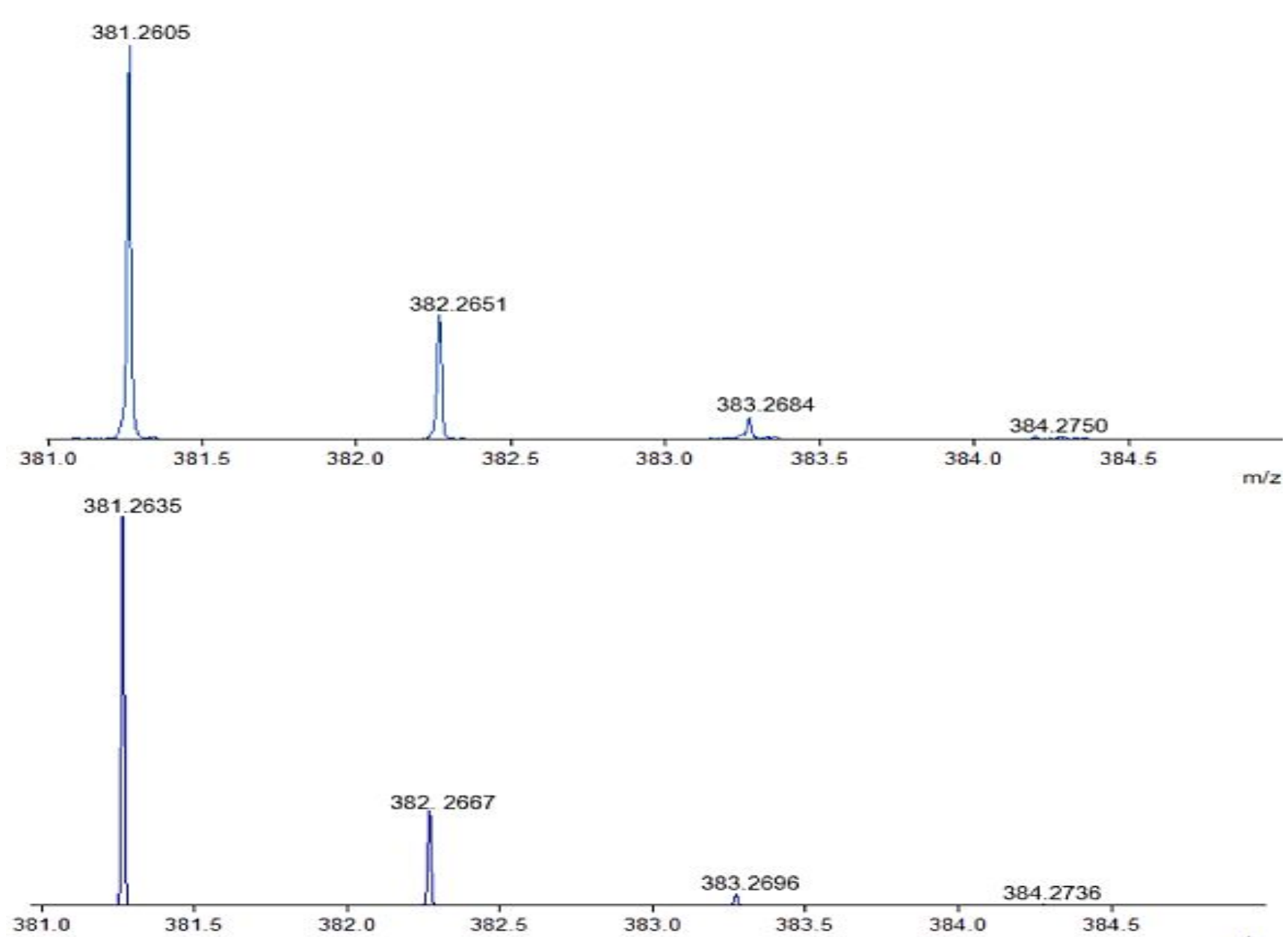

Figure S29. Experimental (top) and calculated (bottom) HR-ESI-Mass Spectra for the [**L6**+D]$^+$ system, in H_2O/CH_3OH (50/50 vol/vol).

Table S1. Crystallographic data for (**1**) and (**2**).

	**1**	**2**
Composition	$C_{11}H_{23}Br_2CuN_6$, Br, H_2O	$C_{22}H_{48}Br_8Cu_2N_{12}$, 4($ClO_4$), 4($H_2O$)
Formula weight / g·mol^{-1}	560.64	1716.94
Size / mm	0.1 × 0.1 × 0.1	0.3 × 0.2 × 0.2
Space group	P 2_1/c	P −1
Unit cell		
a / Å	9.3938(2)	8.2821(5)
b / Å	9.9091(3)	8.5494(4)
c / Å	19.6853(6)	18.9143(9)
α / degrees		93.377(4)
β / degrees	99.464(2)	99.561(4)
γ / degrees		103.936(5)
V / Å3	1807.45(9)	1274.92(12)

Density / g·cm^{-3}	2.06	2.24
Z	4	1
μ / mm^{-1}	7.85	7.39
F000	1100	838
Diffraction Limits	$3.279 < \Theta < 28.283$	$2.985 < \Theta < 29.015$
R(int)	0.0233	0.0482
R(sigma)	0.0306	0.0889
Reflections		
Collected	7911	10949
Unique	4419	5819
Parameters	302	314
Restraints	0	1
*R*1		
total	0.0401	0.1003
$F^2 > 2\sigma(F^2)$	0.0330	0.0683
w*R*2		
total	0.0895	0.2156

$F^2 > 2\sigma(F^2)$	0.0860	0.1869
Goodness of Fit	1.159	1.051
CCDC deposition	2531565	2531564
